# *Bacillus subtilis* DnaB forms multiple protein–protein interactions essential for DNA replication initiation

**DOI:** 10.1093/nar/gkag630

**Published:** 2026-07-02

**Authors:** Aurélie Guyet, Reyes Ruiz Campoy, Petra Manja, Frederic D Schramm, Simone Pelliciari, Stepan Fenyk, Yuanyuan Li, Charles Winterhalter, Aravindan Ilangovan, Heath Murray

**Affiliations:** Centre for Bacterial Cell Biology, Biosciences Institute, Newcastle University, Newcastle Upon Tyne NE2 4AX, United Kingdom; Biodiscovery Institute, School of Pharmacy, University of Nottingham, Nottingham NG7 2RD, United Kingdom; Research Unit in Biology of Microorganisms, Department of Biology, Université de Namur, Namur 5000, Belgium; Centre for Bacterial Cell Biology, Biosciences Institute, Newcastle University, Newcastle Upon Tyne NE2 4AX, United Kingdom; Institute of Medical Biology, Polish Academy of Sciences, Lodowa 106, Łódź 93-232, Poland; Centre for Bacterial Cell Biology, Biosciences Institute, Newcastle University, Newcastle Upon Tyne NE2 4AX, United Kingdom; Centre for Molecular Cell Biology, School of Biological and Behavioural Sciences, Queen Mary University of London, Newark Street, London E1 2AT, United Kingdom; Centre for Bacterial Cell Biology, Biosciences Institute, Newcastle University, Newcastle Upon Tyne NE2 4AX, United Kingdom; Centre for Molecular Cell Biology, School of Biological and Behavioural Sciences, Queen Mary University of London, Newark Street, London E1 2AT, United Kingdom; Centre for Bacterial Cell Biology, Biosciences Institute, Newcastle University, Newcastle Upon Tyne NE2 4AX, United Kingdom

## Abstract

DNA replication is initiated at specific chromosomal loci termed origins. In bacteria, the master replication initiation protein DnaA unwinds the origin (*oriC*), allowing a pair of replicative helicases to be loaded around each strand of the DNA duplex. The molecular mechanisms for managing bacterial helicase loading at *oriC* are unclear. Here we have investigated the role of the essential accessory helicase loader DnaB in *Bacillus subtilis*. By identifying and characterizing DnaB residues that are critical for its role during DNA replication initiation, we have located three necessary protein–protein interactions that DnaB makes with initiation proteins DnaA, DnaD, and DnaI. Combining single particle cryo-electron microscopy, AlphaFold3 predictions, and two-hybrid interaction analyses, the data suggests that DnaB acts as an interaction hub to orchestrate dual helicase loading at the origin. We propose a model for DNA replication initiation in *B. subtilis* and related Firmicutes pathogens that employ DnaB-type helicase loaders.

## Graphical Abstract

**Graphical Abstract:**
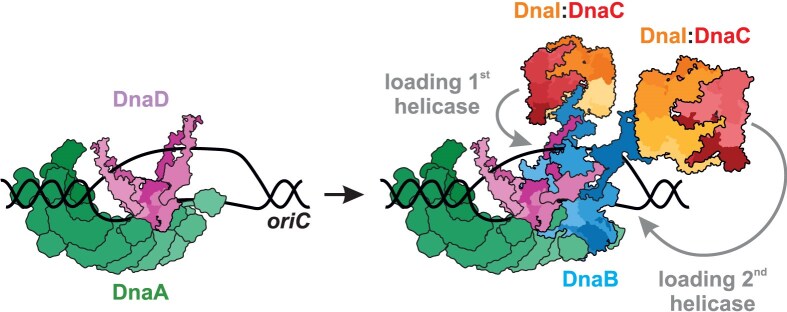

## Introduction

Accurate transmission of genetic material from a parental cell to its progeny is necessary for proliferation. Misregulation of DNA replication initiation can lead to improper chromosome segregation, cell death, and disease [1]. Throughout the domains of life, specific proteins containing related ATPases associated with diverse cellular activities (AAA+) domains (DnaA in bacteria; Orc1/Cdc6 in archaea; and ORC in eukaryotes) assemble into multimeric complexes at chromosome origins and direct loading of two replicative helicases [2–5]. Helicase activity unwinds the origin to allow assembly of two replication machineries that move away from each other, synthesizing new DNA bidirectionally in a template-dependent manner [2].

The stages of bacterial DNA replication initiation are broadly understood. The master initiation protein DnaA binds to specific sites (DnaA-boxes) within the chromosome origin (*oriC*) [6] and assembles into an ATP-dependent oligomer that unwinds the DNA duplex [7–10]. Subsequently, DnaA recruits additional initiation proteins and together this complex enables replicative helicase loading around single-stranded DNA (ssDNA) [4, 11]. Both DnaA and the replicative helicase (a member of the RecA superfamily) are conserved throughout the bacterial domain [12, 13].

While loading the replicative helicase at *oriC* is an essential cell cycle activity, work over the last decade has revealed that, surprisingly, bacterial helicase loaders are not universally conserved [14, 15]. The most well-studied bacterial helicase loaders are members of the AAA+ superfamily, including *Escherichia coli* DnaC [16–18] and bacteriophage λ P protein [19, 20]. Bioinformatic analyses suggest that the AAA+ class of bacterial helicase loaders were originally phage encoded and acquired through horizontal gene transfer [15]. Bacteria lacking AAA+ replicative helicase loaders generally employ members of the DciA or DopE protein families [11].

**Figure 1.**
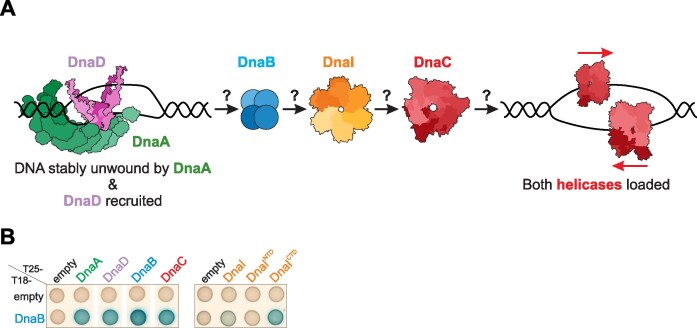
The *B. subtilis* helicase loader DnaB is a protein interaction hub. (**A**) Illustration of the putative *B. subtilis* DNA replication initiation pathway at *oriC*. DnaA (green) oligomerization along the bottom strand enables DNA unwinding. DnaD (pink) is recruited through interaction with DnaA and the single-stranded top strand. In following sequential steps, the helicase loaders DnaB (blue) and DnaI (orange) promote deposition of the two replicative helicases (DnaC, red) onto opposing DNA strands. (**B**) B2H assay showing interactions between DnaB and DnaA/DnaD/DnaB/DnaI/DnaC. Annotations 'T25-' and 'T18-' indicate N-terminal fusions of DNA initiation replication proteins to adenylate cyclase fragments. The 'empty' label indicates the use of the vectors carrying T25- or T18- fragments only (negative control). Strains were spotted onto NA supplemented with X-gal and incubated at 30°C for 48 h (left, 0.008% w/v X-gal) or 72 h (right, 0.016% w/v X-gal).

Beyond containing distinct classes of helicase loaders, some Firmicutes including *Bacillus subtilis, Bacillus anthracis, Enterococcus faecalis, Listeria monocytogenes, Staphylococcus aureus*, and *Streptococcus pneumoniae*, require the additional essential replication initiation factors DnaD and DnaB (not to be confused with the replicative helicase DnaB in *E. coli*) to achieve replicative helicase loading at *oriC* [21–23]. In *B. subtilis*, recruitment of DNA replication initiation proteins required for replicative helicase loading occurs in a linear order (DnaA → DnaD → DnaB → DnaI → helicase DnaC) (Fig. 1A) [24]. In these systems, DnaI is an AAA+ helicase loader [25]. Both DnaD and DnaB assemble into homo-tetramers [21, 22]. Work on DnaD indicates that its essential role during DNA replication initiation is to interact with DnaA and DnaB [26, 27]. The essential activities performed by DnaB are unclear, although the protein has been shown to act as a protein–protein interaction (PPI) hub [27] that can bind various nucleic acid substrates [28–31].

Amino acid residues in *B. subtilis* DnaD necessary for the interactions with DnaA and DnaB have been identified. The N-terminal oligomerization domain of DnaD (DnaD^NTD^) interacts with Domain I of DnaA, while the C-terminal tail (DnaD^CTT^) interacts with DnaB [27, 32, 33]. In the DnaD tetramer, the C-terminal domains (CTDs; DnaD^CTD^) likely extend away from the core of NTDs, allowing the DnaD^CTT^ to specifically recognize a ssDNA element within *B. subtilis oriC* termed the DnaD recognition element (DRE) [28, 34] (Fig. 1A). It has been proposed that during the initiation reaction DnaD stabilizes origin opening and enables recruitment of DnaB [24, 34].

*Bacillus subtilis* DnaB has been proposed to function as a helicase co-loader with DnaI [25]. The N-terminal domain (NTD) of DnaB (DnaB^NTD^) forms the oligomeric core of the protein [21, 30, 35]. It has been reported that DnaB directly interacts with DnaA, DnaD, DnaI and DnaC [25, 27, 32]. However, the molecular basis and physiological relevance of these PPIs remain unclear.

To further understand the function(s) of *B. subtilis* DnaB, here we identified amino acid residues critical for growth and DNA replication initiation, and we provide evidence that these are interaction sites for other essential DNA replication initiation factors. Using a combination of reverse genetics, bacterial two-hybrid (B2H) assays, single particle cryo-electron microscopy (cryo-EM), and structural predictions, we propose a spatial atlas of DnaB PPIs. Based on these findings, a model for DnaB loading a pair of replicative helicases during DNA replication initiation in *B. subtilis* is presented.

## Materials and methods

### Growth conditions and general methods

*Bacillus subtilis* and *E. coli* strains were grown routinely on nutrient agar (NA; Oxoid) and in lysogeny broth (LB: 10 g/l tryptone, 10 g/l NaCl, 5 g/l yeast extract). *Bacillus subtilis* cultures of DnaB variants used for plate reader and spot growth assays, immunoblots and marker frequency analysis were grown in liquid Difco antibiotic medium 3 (Penassay broth or PAB; Oxoid). Detailed culture conditions are provided below.

The following medium supplements were used (unless otherwise stated): ampicillin (100 µg/ml for *E. coli* only), chloramphenicol (5 µg/ml), erythromycin (0.5 µg/ml), kanamycin (5 µg/ml), zeocin (10 µg/ml), spectinomycin (80 µg/ml for *B. subtilis*, 100 µg/ml for *E. coli*), tetracycline (10 µg/ml), 5-bromo-4-chloro-3-indolyl-β-D-galactopyranoside (X-gal 0.008% or 0.016% w/v), xylose (0.5% w/v), and isopropyl-β-D-thiogalactopyranoside (IPTG at 0.1 mM).

Polymerase chain reaction (PCR), plasmid, and genomic DNA (gDNA) were extracted using the QIAquick PCR Purification, QIAprep Spin Miniprep and DNeasy Blood and Tissue kits (Qiagen), respectively.

*Escherichia coli* chemically competent cells were prepared and transformed with DNA via heat shock following the Hanahan method [36]. Candidate colonies were selected on NA supplemented with appropriate antibiotics, propagated in LB with antibiotics at 37°C, screened by colony PCR (if applicable) and purified plasmids were validated by sequencing (detailed below).

*Bacillus subtilis* competent cells were obtained using a two-step starvation procedure [37]. Strains were grown overnight at 37°C in transformation medium composed of Spizizen salts (0.2% w/v ammonium sulphate, 1.4% w/v dipotassium phosphate, 0.6% w/v potassium dihydrogen phosphate, 0.1% w/v sodium citrate dihydrate, 0.02% w/v magnesium sulphate) with supplements (0.5% w/v glucose, 6 mM MgSO_4_, 0.02 mg/ml tryptophan, 0.02% w/v Bacto casamino acids of Gibco, and 1 μg/ml iron[III] ammonium citrate) and inducers (IPTG 0.1 mM or xylose 0.5% w/v) where required. Overnight cultures were diluted 1:20 into 10 ml of fresh transformation medium (with inducer, where required) and grown at 37°C for 3 h with vigorous shaking. An equal volume of pre-warmed starvation medium (Spizizen salts supplemented with 0.5% w/v glucose, 6 mM MgSO_4_ and inducer if required) was added and strains were incubated at 37°C for 2 h with vigorous shaking. Following starvation, DNA was added to 300 μl of competent cells which were further incubated at 37°C with vigorous shaking for at least 1 h. Then, 50–200 μl of each transformation was plated onto selective NA plates supplemented with X-gal (0.008% w/v) where required and incubated at 37°C for 24 h. Resulting strains were validated by streaking colonies on appropriate antibiotic plates and by sequencing PCR products corresponding to genomic loci of interest. Competent cell preparation and transformation procedures were adapted for specific strains to increase transformation efficiency (see details below).

### Oligonucleotides

Oligonucleotides were purchased from Eurogentec to PCR amplify specific loci, assemble recombinant DNA, *in vitro* mutagenesis or to perform quantitative PCR (qPCR) (listed in Supplementary Tables S1 and S2). The original code to generate site-directed mutagenesis primers (for alanine substitution) by an automated program was deposited at Zenodo [26] (https://doi.org/10.5281/zenodo.5541537).

### DNA sequencing and analysis

PCR products and plasmids were sequenced either using Applied Biosystems Big-Dye Ver 3.1 chemistry on an Applied Biosystems model 3730 automated capillary DNA sequencer (University of Dundee, Scotland, www.dnaseq.co.uk) or using Oxford Nanopore Technology (Plasmidsaurus).

SnapGene software (www.snapgene.com), Clone Manager (Sci Ed Software), and the Benchling server (https://benchling.com) were used to engineer genetic modifications (plasmids or genomic) and analyse sequencing results.

### Plasmid and strain construction

Details of plasmid and strain construction can be found in Supplementary Figs S1, S11, S15, and S19, and Supplementary Tables S3–S6.

### B2H assays

Competent cells of the *E. coli* HM1784 strain were co-transformed with two plasmids (20 ng each), one carrying the *Bordetella pertussis* adenylate cyclase T25 domain, the other the complementary T18 domain [38, 39]. Two-hybrid plasmids either carried T18-/T25- domains on their own (labelled *empty*) or fused to the 5′-end of genes of interest (Supplementary Table S4). After a heat shock of 90 s in a 42°C water bath, cells were incubated on ice for at least 5 min. Each transformation was resuspended in 950 µl of LB supplemented with ampicillin and spectinomycin (80 µg/ml), then transferred into a 15 ml Falcon tube and incubated with shaking at 37°C for 23–24 h. Samples were transferred into sterile 96-well microplates (Corning, 353 072) and diluted 1:100 in LB prior to spotting 5 µl onto NA plates with ampicillin (100 µg/ml), spectinomycin (100 µg/ml), and X-gal (0.008% or 0.016% w/v, see figure legends) using a multichannel pipette. Plates were incubated at 30°C for 2–3 days and scanned. Experiments were done at least twice, and representative data are shown.

Positive interaction is observed by the formation of blue colonies, which indicates that cyclic AMP has been produced. Note that uneven blue pigmentation was initially observed at the outer edges of peripheral spots after prolonged plate incubation. To prevent this, subsequent B2H assays were performed with the set of spots of interest surrounded by bacterial “buffer” spots (e.g. experimental sets are framed by negative interaction spots).

### Plate imaging and processing

Plates were scanned with an Epson perfection V800 photo scanner, applying specific settings for B2H assays (in *image adjustment* settings: brightness 20, contrast 15, saturation 10, and yellow 20). Images were cropped with Fiji (ImageJ) software [40] where necessary.

In figures, all spots within a single, black-framed image originate from the same plate. White lines are included to facilitate visualization (e.g. boundaries between residue numbers).

### Automated plate reader analyses

A single fresh colony per well (control strains AG02 and CW53, and 42 DnaB variants) was used to inoculate 900 µl of PAB with 0.1 mM IPTG in a sterile 96-deepwell plate (Agilent, 201240-100). Plates were sealed with Breathe-Easy membranes (Diversified Biotech BEM-1, Z380059) and incubated at 37°C on SciQuip microplate mixers at 900 rpm for ∼22 h.

Overnight cultures were diluted in a two-step serial dilution (1:32 twice, resulting in a final ∼ 1:1000 dilution) within sterile 96-well microplates (Corning, 353 072). Intermediate dilutions in PAB only were used to inoculate a second microplate, where each strain was further diluted in PAB in the absence or presence of IPTG (0.1 mM).

The final microplate was sealed with a new Breathe-Easy membrane and placed in a pre-warmed Sunrise microplate reader (Tecan). Cell growth was monitored at 37°C with high intensity shaking setting, recording the absorbance at 600 nm (A_600_) every 6 min for 22 h. Data were collected using Magellan software (version 7.2). At least two biological replicates were performed independently for each strain. Microsoft Excel was used for data analysis and GraphPad Prism version 10 to generate graphs. One representative dataset is shown, focusing on the 2–8 h growth period (raw data in Supplementary Table S7).

### Spot-titre analysis of *B. subtilis* strains

To analyse the growth of strains with DnaB and DnaI variants, cells were grown overnight (18–20 h) in test tubes containing PAB with IPTG (0.1 mM) at 30°C with vigorous shaking. One millilitre of culture was collected, washed twice in PAB, and normalized to an A_600_ of ∼0.3. Cells were transferred to a sterile 96-well microplate (Corning, 353 072) and serially diluted 1:10 in PAB. A multichannel pipette was used to spot 5 μl of each dilution onto PAB agar plates in the presence or absence of IPTG (0.1 mM).

For DnaA variants, overnight growth was performed in LB with xylose, cells were washed and diluted in LB only, and 4 μl spots of serial dilutions were applied onto NA plates in the absence or presence of xylose (0.5% w/v).

Plates were incubated at 37°C and scanned on different days. Spot titre assays were repeated independently at least twice and representative data is shown.

### Collection and preparation of soluble proteins

Cultures were grown overnight at 30°C in PAB in presence of IPTG (0.1 mM, for DnaB and DnaI variants) or in LB supplemented with xylose (0.5% w/v, for DnaA variants). Cultures were diluted to an A_600_ of ∼0.04 in 18 ml of pre-warmed supplemented medium (same as the overnight composition) and grown at 37°C with shaking until they reached an A_600_ of ∼0.3. Cells (12 ml) were harvested using a swing centrifuge (3273 × *g*, 5 min) at room temperature, washed in pre-warmed medium, spun down, and resuspended in 12 ml of medium without inducer. Cells (5 ml) were transferred to a test tube (in duplicate, if an inducer sample condition was required) and incubated at 37°C for 90 min with shaking. Samples (800 μl) were collected, spun down briefly, and cell pellets were flash frozen in liquid nitrogen.

For experiments with samples collected at different time points after inducer withdrawal, the following steps were adjusted: overnight cultures were diluted into 30 ml and cells were resuspended in an equal volume without inducer. The entire culture was transferred to a flask, incubated at 37°C with shaking, and samples were collected every 15 min up to 2 h following inducer withdrawal (time 0).

Cell pellets were resuspended in 180 μl of lysis buffer (10 ml of 100 mM Tris–HCl [pH 7.4] containing one cOmplete Mini ethylenediaminetetraacetic acid (EDTA)-free protease inhibitor cocktail tablet from Roche). Samples were sonicated in a 4°C room using a Sonics Vibra-Cell^TM^ VC 130 ultrasonic processor fitted with a probe of 2 mm tip diameter using two cycles of 8 × 3 s pulses at 40 amplitude with incubation on ice (at least 10 min) between repetitions. Samples were then normalized to their A_600_ at the time of collection and prepared with NuPAGE LDS sample buffer (Life Technologies) in the presence of 100 mM reducing agent dithiothreitol (DTT). Samples were heated at 70°C for 10 min and spun down in a centrifuge before loading supernatants.

### Sodium dodecyl sulphate–polyacrylamide gel electrophoresis and immunoblotting

Total protein samples were separated by electrophoresis using NuPAGE Bis–Tris Midi 8% gels (Invitrogen) in chilled NuPAGE MOPS sodium dodecyl sulphate (SDS) buffer with NuPAGE antioxidant (Invitrogen) for ∼100 min at 180 V, including a Prestained Protein Ladder (Abcam, ab116027) for reference. The midi gel was transferred to a 0.45 μM Amersham Hybond P polyvinylidene difluoride (PVDF) methanol-activated membrane (GE Healthcare) using a semi-dry Trans-Blot Turbo transfer system (Bio-Rad), Wypall X60 cloths, and transfer buffer (600 mM Tris, 600 mM glycine, 280 mM Tricine, 2.5 mM EDTA, and 0.05% SDS). After standard MIDI-transfer, the membrane was washed twice for 5 min in PBS-T (phosphate-buffered saline, Oxoid + 0.1% Tween 20, Sigma) on a rocking shaker (shaking applied to all subsequent steps, excluding detection). The membrane was then blocked in PBS-T with 7% w/v milk (PBS-T-milk) overnight at 4°C.

The membrane was incubated with fresh PBS-T-milk for 1 h at room temperature with relevant polyclonal rabbit antibodies: anti-DnaB (1:2000), anti-DnaA (1:2000), and anti-DnaI (1:1500). As loading control, a polyclonal rabbit anti-FtsZ antibody (1:10 000) was co-incubated alongside the other primary antibody. For DnaB/FtsZ experiments, the membrane was cut above the 41 kDa marker after blocking, allowing for protein detection with individual antibodies. After 1 h, the membrane was washed four times with PBS-T, then incubated for another hour with PBS-T-milk containing an anti-rabbit IgG-peroxidase conjugated antibody (1:10 000) (A6154, Sigma–Aldrich). The membrane was then washed four times with PBS-T and incubated for 5 min with Clarity Western ECL Substrate (Bio-Rad). Chemiluminescence was detected using an ImageQuant LAS 4000 imager (GE Healthcare) and images edited in Fiji (ImageJ). At least two biological repeats were performed and representative images are shown.

Laboratory antibodies mentioned above were manufactured by Eurogentec and used in previous studies [34, 41]. Note that immunoblots showed a reproducible slower protein migration pattern for DnaB L315D, DnaI F131A/D, and DnaI V134A/D variants.

### Marker frequency analysis using qPCR

Strains were grown overnight at 30°C in PAB in presence of IPTG (0.1 mM). Cultures were diluted to an A_600_ of ∼0.03 in a final volume of 5 ml of pre-warmed PAB containing IPTG (0.1 mM) and grown with shaking at 37°C until A_600_ reached ∼0.3. Cells were pelleted, washed twice in pre-warmed PAB, resuspended and diluted six-fold into 5 ml of pre-warmed PAB, before being incubated for 60 min at 37°C. Samples (1 ml) were collected and vortexed with sodium azide (0.05% w/v final) to arrest cell metabolism. Subsequently cell pellets were collected by centrifugation and flash frozen in liquid nitrogen. gDNAs were extracted and eluted in 100 µl of UltraPure DNase/RNase Free water (Invitrogen) prior to 1:100 dilutions for qPCR analysis.

The relative amount of DNA near the chromosome origin (amplified using qSF11: 5′-GGGGATCAATCGGGGAAAG-3′, qSF12: 5′-AGTAGGGCCTGTGGATTTG-3′, efficiency 90%, R^2^ = 0.99888) and terminus (amplified using qPCR57: 5′-TTTGCATGAACTGGGCAATA-3′, qPCR58: 5′-TCCGAACATGTCCAATGAGA-3′, efficiency 88%, R^2^ = 0.99903) was determined by qPCR. A QIAgility robotic workstation (Qiagen) was used to automatically dispense 6 µl of diluted gDNA and 14 µl of a pre-mixed solution (containing 10 µl of Luna Universal qPCR mix from NEB and 2 µl of each primer at 10 µM final) into a Rotor-Disc 100 (Qiagen). qPCRs were run on a Rotor-Gene Q real time PCR cycler (Qiagen) with the following program: 1 cycle of 3 min at 95°C, followed by 40 cycles of 15 s at 95°C and 30 s at 60°C. A melt curve was performed at the end of each run to confirm amplification specificity. Negative controls using UltraPure water instead of gDNA were included in each run to rule out contamination. The critical threshold cycle (C_T_) was defined for each sample using the Rotor-Gene Software version 2.3.5 (Qiagen). For each strain, *ori*/*ter* ratios were averaged from 1/2^CT^ values of two technical replicates. Data were normalized to the *ori:ter* ratio of spore gDNA included within the same run. Graphs show average and standard error corresponding to four independent biological repeats. C_T_ values for each reaction and statistical analysis are provided (Supplementary Table S8).

### Statistical analysis

Statistical analyses were performed using the GraphPad website (https://www.graphpad.com/quickcalcs/ttest1/). Significance was determined using unpaired, two-tailed Student’s *t*-tests to determine *P*-values comparing DnaB variants to wild-type DnaB (reference). *P*-values obtained are provided in Supplementary Tables S8 and S9, along with mean ratio, standard deviation, and number of biological replicates (indicated in relevant supplementary tables and methods).

### Fluorescence microscopy and cell counting

Strains were grown in chemically defined medium (Spizizen salts supplemented with 0.1% w/v glutamate, 0.02 mg/ml tryptophan, 6 mM magnesium sulphate, 0.13 mM manganese sulphate, 0.1 mM calcium chloride, 1 μg/ml iron[III] ammonium citrate, 0.5% w/v glycerol, 0.02% w/v Bacto casamino acids) supplemented with IPTG (0.1 mM), and incubated with shaking at 37°C overnight. Cultures were diluted to an A_600_ of ∼0.02 in 20 ml of pre-warmed chemically defined medium without casamino acids in the presence of IPTG (0.1 mM) and grown with shaking up to an A_600_ of ∼0.1 at 37°C. Cells (14 ml) were collected by centrifugation, washed, resuspended in an equal volume of pre-warmed chemically defined medium (without IPTG and casamino acids), and incubated with shaking at 37°C. Cells were imaged 90 min following inducer withdrawal. Samples (0.5 µl) were immobilized onto a thin layer of 1.4% w/v agarose diluted in sterile ultra-pure water using Teflon-coated multispot microscope slides (Thermo Fisher). Samples were allowed to dry, covered with a glass coverslip (VWR) and imaged immediately at 37°C using an Nikon Eclipse Ti microscope equipped with a CoolLED pE-300 white light source, a Photometrics Prime camera, a Nikon Plan Apo 100×/1.40 Oil Ph3 objective, and fluorescence filter sets for GFP (Chroma 49 002, EX470/40, DM495lpxr, EM525/50) and mCherry detection (Chroma 49 008, EX560/40, DM585lprx, EM630/75). Images were acquired with the NIS elements software v5.42 (Nikon).

Image analyses were performed using Fiji software version 2.16 [40]. The ObjectJ plugin (version 1.05n, https://sils.fnwi.uva.nl/bcb/objectj/) was used to manually count the number of origins, nucleoids and cells from composite images. Composite images were generated by merging background-subtracted mCherry/GFP signals with phase contrast images, pre-processed with default *Find Edges* and *Smooth* menu commands to enhance cell outlines. This processing workflow was applied consistently to all frames. At least two biological repeats were performed independently for each strain (*n* ≥ 2), with a minimum of 200 cells analysed per experiment. For each strain, values for mean ratio, standard error, and statistical significance (*P*-values) are provided (Supplementary Table S9). Data were processed in Excel and graphs generated in GraphPad Prism 10.

Representative images shown were processed using Fiji software. Overlay images were generated by merging three channels: background-subtracted GFP channel, cell outlines extracted from phase contrast images (grey channel), and mCherry channel pre-processed to facilitate origin foci visualization. A binary mask of peak fluorescence signal was created using the *Process > Find Maxima* menu command (Prominence >300, output single point) and enlarged to a 2-pixel radius spot via *Process > Filters > Maximum*.

### DnaB purification

A plasmid encoding for His_14_-SUMO-DnaB (pSF15) was transformed into *E. coli* BL21(DE3)-pLysS. Cells harbouring the plasmid were grown in 2× YT medium (16 g/l tryptone, 10 g/l yeast extract, 5 g/l NaCl) [42] at 37°C and at A_600_ ∼0.4, cultures were supplemented with 1 mM IPTG and further incubated for 4 h at 30°C. Cells were harvested by centrifugation at 7000 × *g* for 20 min, resuspended in 40 ml of Ni^2+^ Binding Buffer (40 mM Tris–HCl [pH 8.0], 50 mM sodium chloride, 30 mM imidazole) containing one EDTA-free protease inhibitor tablet (Roche, #37 378 900) and flash frozen in liquid nitrogen. Cell pellet suspensions were thawed and incubated with 0.5 mg/ml lysozyme with agitation at 4°C for 1 h. Cells were disrupted by sonication (Fisherbrand 505, 60 min of 20 s ON/OFF cycles at 30% power with a ¼ inch tip in an ice bath). To remove cell debris, the lysate was centrifuged at 24 000 × *g* for 30 min at 4°C, then passed through a 0.2 μm filter for further clarification. All further purification steps were performed at 4°C using fast protein liquid chromatography (FPLC) with a flow rate of 1 ml/min.

The clarified lysate was applied to a 1 ml HisTrap HP column (GE), the column washed with 10 ml Ni^2+^ High Salt Wash Buffer (40 mM Tris–HCl [pH 8.0], 2 M sodium chloride, 30 mM imidazole) and 10 ml of 10% Ni^2+^ Elution Buffer (40 mM Tris–HCl [pH 8.0], 50 mM sodium chloride, 1 M imidazole). Proteins were eluted with a 10 ml linear gradient (10%–100%) of Ni^2+^ Elution Buffer. Fractions containing the protein were applied to a 1 ml Mono-Q 5/50 GL affinity column (GE) equilibrated in Q Binding Buffer (40 mM Tris–HCl [pH 8.0], 50 mM sodium chloride). Proteins were eluted with a 20 ml linear gradient (0%–50%) of Q Elution Buffer (40 mM Tris-HCl [pH 8.0], 2 M sodium chloride). Fractions containing DnaB were pooled and digested overnight with 10 μl of 10 mg/ml His_14_-TEV-SUMO protease [43].

The digested reaction salt was adjusted to 50 mM sodium chloride based on the Mono-Q conductivity value and the product was applied to a 1 ml HisTrap HP column pre-equilibrated in Q binding buffer to capture uncleaved protein, His_14_-SUMO-tag, and His_14_-TEV-SUMO protease. The flow-through of the affinity column was concentrated with Amicon Ultra 10 kDa cutoff (Sigma–Aldrich), glycerol was added (20% w/v final) and protein aliquots flash frozen in liquid nitrogen before storage at −80°C.

Fraction eluates were separated by electrophoresis using a NuPAGE 4%–12% Bis–Tris gradient gel run in NuPAGE MES SDS buffer (Life Technologies) alongside a Novex Sharp Pre-stained Protein Standard (Invitrogen). Proteins within the gel were visualized using InstantBlue staining (Merck).

### Sample preparation for single particle cryo-EM analysis

A 4 µl sample of purified wild-type full-length DnaB was applied to plasma-cleaned Quantifoil Au 2/2 200-mesh grids and plunge-frozen in liquid ethane using a Leica EM GP2 plunger. Cryo-EM data were collected at liquid-nitrogen temperature on a Titan Krios transmission electron microscope (Thermo Fisher Scientific) at the London Consortium for Electron Microscopy (LonCEM) operated at 300 keV. Micrograph movies were acquired using EPU software (FEI) on a Gatan K3 BioQuantum detector in counting mode, with a calibrated pixel size of 0.85 Å. A total of 12 516 movies were collected over a defocus range of approximately −0.7 to −3.0 µm. Each movie comprised 36 frames, corresponding to a total electron dose of ∼40 e⁻/Å² at a dose rate of 15 e⁻/pixel/s.

### Cryo-EM image processing and reconstruction

The movie stacks were aligned and summed using patch MotionCor2 [44] using Relion [45] via the on-the-fly processing pipeline at (LonCEM). The motion-corrected micrographs were imported into cryoSPARC (v4.7.1) [46] and micrograph CTF was estimated using the patch CTF estimation function. After screening the micrographs for good Thon Rings and ideal ice thickness, 12 516 micrographs remained in the dataset for further processing. The blob picking tool was used to pick an initial set of 73 617 particles from a subset of 122 micrographs that was classified to generate the initial templates for subsequent rounds of picking. Once the right templates and picking parameters were obtained, a total of 2276 865 particles were picked from the entire dataset and extracted using a box size of 256 × 256 with two-fold binning. Low-quality particles were removed after several iterative rounds of 2D classification in cryoSPARC, resulting in a final stack of 385 897 particles. These particles were used for two different purposes: to generate higher quality 2D templates for improved initial model generation; and to curate DnaB particles with the best features for downstream refinement.

The set of 385 897 particles was used for 3D classification into 10 classes, applying a filter resolution of 6 Å, aiming to curate the best DnaB particles. A total of 144 168 particles from the 3D classification step were re-extracted without binning and subjected to homogeneous refinement. The output from one of the ab initio classes, which showed more clearly defined map features, was used as the reference. This yielded a 3.25 Å reconstruction. This map was further refined by nonuniform refinement imposing C2 symmetry, and including per-particle defocus, Ewalds sphere (EWS) correction, as well as optimized CTF parameters. The final refinement resulted in a 3.1 Å reconstruction for DnaB. This map was used for model building. Statistics for data collection and 3D refinement are included in Supplementary Table S10.

### Model building and refinement

Model building started by identifying the four DnaB domains for reconstruction. An AlphaFold model obtained through AlphaFold3 (AF3) [47] including four copies of DnaB was used as starting point for modelling. This model contained two DnaB^CTD^ regions that were not present in the map, hence they were deleted prior to rigid-body fitting into the cryo-EM map. Once a starting model was obtained, a Gaussian filter was applied to the density on ChimeraX to facilitate interpretation of DnaB secondary structure elements. This allowed a stepwise fitting of DnaB helices and loops into the density using the Fit to Map function on ChimeraX [48]. Closer inspection in Coot [49] revealed incorrectly built regions which were manually rebuilt using Coot and a Ramachandran outlier-free initial model was generated to be input into real space refinement. The outlier-free model was used for real space refinement and subsequent Coot model building to correct for geometry, density fit, and rotamer accuracy. This model was subjected to several iterative rounds of real space refinement using Phenix [50] and modification in Coot while progress was monitored using Ramachandran plot and Molprobity [51]. Cryo-EM data processing and model refinement software are compiled and supported by the SBGrid Consortium [52].

### AlphaFold3 and protein representation

AF3 server [47] was used to predict both protein folding and protein–protein interfaces. Predicted aligned error (PAE) heatmaps for the AF3 models were generated using the PAE Viewer (https://pae-viewer.uni-goettingen.de) [53]. The output structural models were individually analysed in UCSF ChimeraX for overall fold quality, domain organization, and interface geometry [48].

The hybrid tetrameric DnaB model was generated by combining the experimentally derived cryo-EM reconstruction with AF3 predictions. In the cryo-EM model, residues 7–171 were resolved in chains A and D, corresponding to the NTD, whereas residues 7–289 were resolved in chains B and C, corresponding to the NTD plus middle domain (MD). The cryo-EM reconstruction did not resolve the CTD or the CTT. To complete the model, an AF3 prediction of the DnaB tetramer was generated (Supplementary Fig. S7). Individual chains were then isolated and fitted into the cryo-EM assembly. For chains A and D, residues 172–472 were fitted. For chains B and C, residues 290–472 were fitted. Added regions were manually adjusted in Coot [49] to remove steric clashes within the tetramer. This procedure yielded the final hybrid tetrameric DnaB model shown in Figs 4C–E and 8A, and Supplementary Fig. S7E–G.

### Alignment of protein homologs from selected pathogens

Protein sequences were sourced from *Subti* Wiki [54], Uniprot [55], NCBI [56], BioCyc for *E. faecalis* OG1RF [57], *Pneumo* Wiki [58], and PneumoBrowse 2 for *S. pneumoniae* D39V [59] and are listed in Supplementary Table S11. Multiple protein sequence alignments were obtained using the Clustal Omega server [60]. Full-length protein sequences of DnaB, DnaA, DnaD, and DnaI homologs from selected pathogens (*B. anthracis, E. faecalis, L. monocytogenes, S. aureus, S. pneumoniae*) were aligned to *B. subtilis* proteins with input data available in Supplementary Tables S12, S13, S14, and S15, respectively.

## Results

### DnaB is an interaction hub for DNA replication initiation proteins

Our aim was to dissect *B. subtilis* DnaB PPIs using a B2H assay [38]. Here a Δ*cya* deletion strain of *E. coli* is used as a heterologous host to express two proteins of interest. Test genes are fused to fragments of *Bordetella pertussis* adenylate cyclase (T18 or T25) encoded on compatible multicopy plasmids. A positive PPI is detected when the two proteins of interest are in close proximity, bringing the T25 and T18 peptides together to reconstitute a functional adenylate cyclase enzyme. The resulting cyclic AMP production activates expression of the endogenous *lacZYA* operon, which can be detected on solid growth medium using the chromogenic substrate X-gal.

Because expression of *B. subtilis* DNA replication initiation proteins in *E. coli* can be toxic [25, 27, 61, 62], a modified *E. coli* reporter strain that lacks the *rnhA* gene (encoding for RNase HI) was employed [32]. It is thought that R-loops accumulate in the absence of RNase HI [63], providing a secondary avenue for the cell to initiate DNA replication, thereby decreasing the interference caused by heterologous DNA replication initiation proteins.

Full-length *dnaA, dnaD, dnaB, dnaI*, and *dnaC* genes were fused to either T18 or T25 fragments, and all proteins were tested against DnaB in the reporter strain. This benchmark analysis showed that DnaB interactions could be detected with all other DNA replication initiation factors, including itself (Fig. 1B). The PPI between DnaB and full-length DnaI appeared relatively weak; therefore, to enhance the signal, we tested the individual NTDs and CTDs of DnaI. A stronger interaction was observed between DnaB and DnaI^CTD^ (Fig. 1B), consistent with a previous study [27]. These results indicate that the B2H assay is suitable and specific for analysis of DnaB PPIs with other DNA replication initiation proteins.

### Identification of critical residues in DnaB required for DNA replication initiation

**Figure 2.**
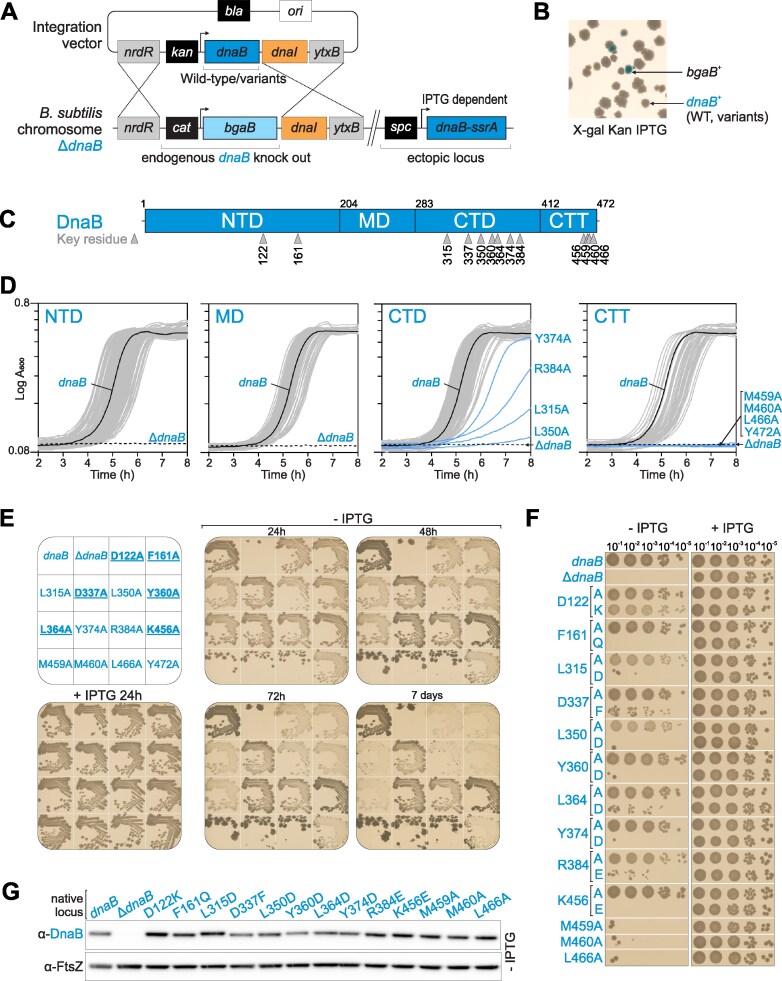
Identification of critical residues in *B. subtilis* DnaB. (**A**) Illustration of the genetic pipeline employed to construct the DnaB alanine substitution library. An integration vector (or its derivative) carrying *dnaB* (wild-type or variant) replaces *bgaB* at the endogenous locus of a Δ*dnaB* strain. This strain also contains an ectopic IPTG-inducible degron tagged *dnaB* (*dnaB–ssrA*) to conditionally complement alleles at the endogenous locus. (**B**) Example of a NA plate supplemented with kanamycin, X-gal (0.008% w/v), and IPTG (0.1 mM) obtained after transformation of *B. subtilis* Δ*dnaB* with an integration vector. (**C**) DnaB primary structure indicating the NTD (NTD 1–203), MD (MD 204–282), CTD (CTD 283–411), and C-terminal tail (CTT 412–472). Critical DnaB residues are indicated. (**D**) Growth assay of the 447 DnaB alanine variants in liquid medium lacking IPTG. Absorbance measurements were taken over time and the data is shown across four plots based on the DnaB domains. Black lines show the growth profile of wild-type *dnaB*, dashed black lines show the Δ*dnaB* strain, grey lines show DnaB variants with a wild-type growth profiles, and blue lines show DnaB variants with a growth defect (residue number indicated). (**E**) A total of 14 DnaB alanine variants displayed mild to significant change in colony morphology phenotypes on a PAB agar plate lacking IPTG. Variants identified on solid medium are indicated in bold and underlined. Wild-type and Δ*dnaB* strains are included as controls (top left). Plates were incubated at 37°C and scanned at indicated timepoints. (**F**) Spot titre assay showing growth of strains harbouring DnaB amino acid substitutions after 24 h at 37°C (including variants from Fig. 2D-E). (**G**) Immunoblot of DnaB variants following depletion of DnaB–ssrA for 90 min. The tubulin homolog FtsZ was used as a standard. Strains are the same as used in panel (F).

We hypothesized that one or more DnaB PPIs would be critical for cell viability. To identify amino acids critical for DnaB function in *B. subtilis*, an alanine scan was performed. Using a plasmid integration vector that targets the endogenous *dnaB* operon (Fig. 2A and Supplementary Fig. S1), a library of 447 *dnaB* mutants was constructed, each encoding for a single alanine substitution along the protein (excluding the first codon and any endogenous alanine). Library plasmids were individually transformed into a *ΔdnaB* strain carrying an ectopic inducible copy of *dnaB* tagged with an *ssrA* degron motif (Fig. 2A and Supplementary Fig. S1) [32, 64, 65]), and depletion of DnaB–ssrA following inducer withdrawal was confirmed (Supplementary Fig. S2A). To ensure correct plasmid integration at the endogenous *dnaB* locus, the recipient strain also contained *bgaB* (encoding β-galactosidase) at the endogenous *dnaB* locus, allowing chromogenic detection with X-gal (Fig. 2B). Transformants were selected and propagated in the presence of IPTG to express the ectopic DnaB–ssrA, before being grown in the absence of inducer to determine the consequence of each DnaB alanine substitution.

For initial phenotypic analyses, strain growth was assessed in liquid medium using a plate reader. Absorbance measurements were plotted as a function of time and data are shown for each DnaB domain (Fig. 2C and D). Control strains show the growth of wild-type *dnaB* (solid black) and the Δ*dnaB* mutant (dashed black) (Fig. 2D). Using this approach, eight DnaB alanine variants displayed a growth defect (shown in blue).

Growth of DnaB alanine substitutions was also assessed on solid medium. In addition to the mutants reported above, six alanine variants were identified based on their altered colony colour or reduced cell density compared to the strain with wild-type *dnaB* (Fig. 2E, bold and underlined). To potentially exacerbate the defects caused by this set of mutants, more severe DnaB amino acid substitutions were constructed. These mutants either reverse the charge of a residue or swap hydrophobic/polar side chains. Strains were serially diluted and spotted onto solid medium with or without IPTG. The results showed that all further substitutions (except for D122K) caused a loss in colony forming units (Fig. 2F). DnaB^D122A^ and DnaB^D122K^ variants produced colonies with abnormal morphology, with the latter exhibiting a sectored phenotype with additional growth atop the primary colony, reflecting stress-induced heterogeneity (Supplementary Fig. S3). Immunoblots confirmed that all but one of the DnaB variants are stably expressed following depletion of DnaB–ssrA; the exception was DnaB^Y472A^ which was not analysed further (Fig. 2G, and Supplementary Fig. S2B–E). Collectively, these analyses identify thirteen critical DnaB residues whose substitution disrupts growth or colony morphology (Fig. 2C).

**Figure 3.**
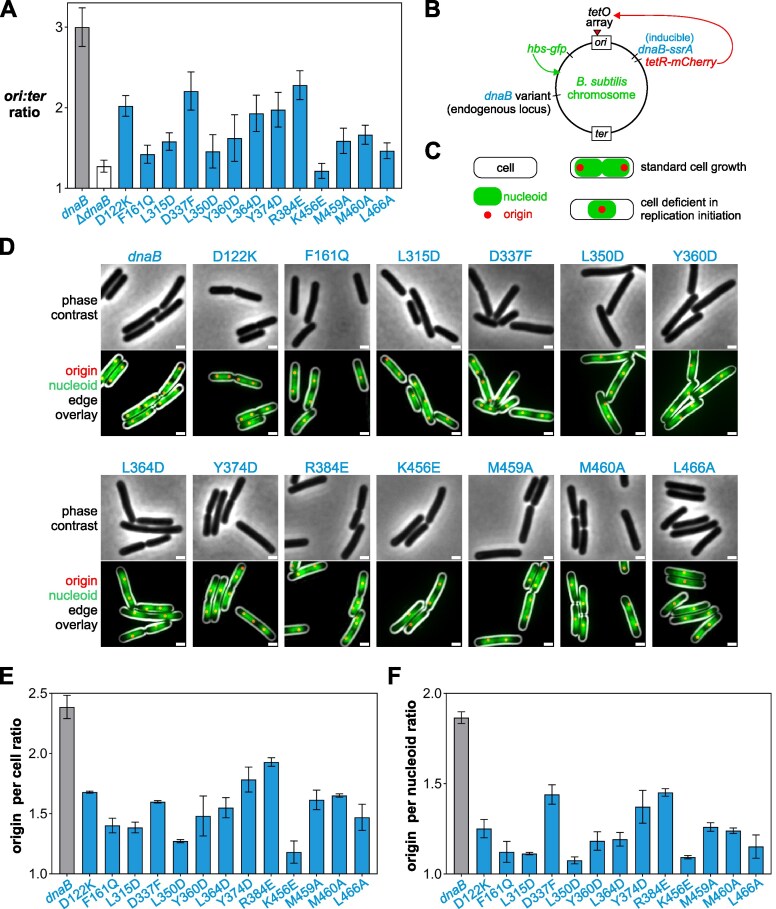
DnaB variants are defective in DNA replication initiation. (**A**) Marker frequency analysis of DnaB variants was determined using qPCR. The strains are identical to those in Fig. 2F and G. gDNA was isolated from exponentially growing cells following depletion of DnaB–ssrA for 60 min. Data were normalized to gDNA of spores. Error bars represent the standard error of the mean from four biological replicates (*n* = 4). Statistical significance was determined using a two-tailed unpaired Student’s *t*-test (*P*-values are provided in Supplementary Table S8). (**B**) Illustration of the fluorescence labelling system used to visualize the chromosome origin (TetR-mCherry binds a *tetO_48_*, red) and the nucleoid (Hbs-GFP, green). (**C**) Expected fluorescence patterns for replicating and non-replicating cells. (**D**) Representative microscopy images of the fluorescent reporter strain harbouring the different DnaB variants. Data were acquired from live exponentially growing cells following depletion of DnaB–ssrA for 90 min. Scale bar is 1 µm. (**E, F**) Quantification of *oriC* number per cell (**E**) and nucleoid (**F**). Error bars represent the standard deviation of the mean for at least two biological replicates (*n* ≥ 2), with a minimum of 200 cells counted per experiment. Statistical significance was determined using a two-tailed unpaired Student’s *t*-test (*P*-values are provided in Supplementary Table S9).

DnaB is essential for DNA replication initiation both at the chromosome origin and at a repaired replication fork [66]. To determine whether the identified amino acid substitutions in endogenous DnaB perturb *oriC*-dependent initiation, two complementary approaches were used, both following depletion of the ectopically expressed DnaB–ssrA. First, marker frequency analysis was used to determine the frequency of DNA replication initiation at a population level. Following chromosomal DNA extraction, qPCR analysis showed that all DnaB variants displayed a lower *ori:ter* ratio compared to wild-type (Fig. 3A), consistent with a decreased rate of replication initiation. Second, epifluorescence microscopy was used to determine the frequency of DNA replication initiation at the single cell level. In a reporter strain that harbours *hbs-gfp* for chromosome visualization (green) [67] and a fluorescent reporter-operator system for *oriC* labelling (red) (Fig. 3B) [32, 68], wild-type *B. subtilis* characteristically displays two origins per nucleoid (Fig. 3C) [68, 69]. However, a cell deficient in DNA replication initiation typically contains a single origin located near the centre of a chromosome mass [32, 70]. Representative cells encoding *dnaB* alleles, imaged 90 min after DnaB–ssrA depletion, are displayed in Fig. 3D. The results showed that all DnaB variants displayed fewer origins per cell and nucleoid (Fig. 3E and F), again consistent with a replication initiation defect.

Based on these genetic, biochemical, and cellular results, 13 DnaB residues were identified as critical for replication initiation from *oriC*. To investigate the potential role(s) of these key residues, we sought to determine the molecular structure of DnaB.

### A new domain architecture for *B. subtilis* DnaB reveals critical residue clusters

**Figure 4.**
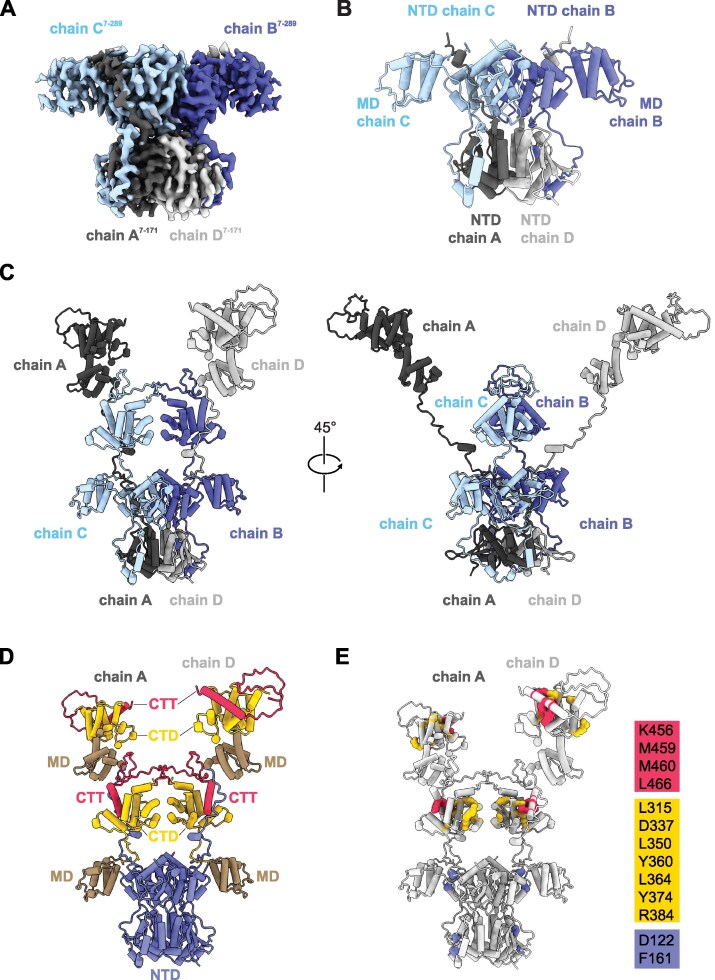
Cryo-EM structure and hybrid model of DnaB tetramer. (**A**) Cryo-EM map of the DnaB tetramer. The four chains are individually coloured (A in dark grey, B in purple, C in blue, and D in light grey). (**B**) Model of the cryo-EM DnaB tetramer shown in cartoon representation, coloured based on the individual chains. Only the NTD (all chains) and MD (two chains) are visible. (**C**) A hybrid model combining cryo-EM data and AF3 structural predictions. The complete DnaB tetramer is coloured based on individual chains, with a 45° y-axis rotation represented. (**D**) Hybrid model of the DnaB tetramer with the domains individually coloured in all chains. (**E**) The hybrid model of DnaB highlighting key residues (listed on the right) coloured according to the domain in which they reside.

To elucidate the structural basis for DnaB function, DnaB was purified (Supplementary Fig. S4) and its structure determined using single-particle cryo-EM (Fig. 4, Supplementary Fig. S5, and Supplementary Table S10). Initial cryo-EM image analyses revealed distinct structural features at the 2D classification level, and subsequent 3D refinement consistently indicated twofold symmetry (Supplementary Fig. S5A). Accordingly, the final reconstruction was refined with C2 symmetry, yielding a cryo-EM density map at 3.1 Å (Supplementary Fig. S5B–D) that accommodates four DnaB protomers (Fig. 4A).

The cryo-EM structure of DnaB reveals a tetramer with clear two-fold symmetry (Fig. 4A and B, and Supplementary Fig. S6A-C). Within the assembly, chains A and D are resolved up to residue 171, whereas chains B and C extend to residue 289 (Fig. 4B). Thus, chains B and C contain the NTD, the complete linker connecting the NTD to the MD, and the MD itself (residues 7–289; Fig. 2C). Continuous density is visible between the NTD and MD in chains B and C. This density extends from each NTD, wraps around the NTDs of chains A and D, respectively, and then connects to the MDs of chains B and C, thereby positioning each MD adjacent to the NTD core (Supplementary Fig. S6A).

By contrast, chains A and D are composed predominantly of the NTD, with only partial density for the NTD to MD linker, while the MD is not visible in the map (residues 7–171; Fig. 4A and B). In both chains, the density extending beyond residue 171 projects away from the NTD core, unlike the corresponding regions in chains B and C, where the linker returns towards the core and connects to the MD (Supplementary Fig. S6A and B). These distinct conformations adopted by the two pairs of subunits give rise to the pronounced two-fold symmetry of the tetramer (Fig. 4B). This feature is particularly evident when the structure is compared with the X-ray crystal structure of the closely related DnaB homologue from *Geobacillus stearothermophilus* (Supplementary Fig. S6D-E). In the *G. stearothermophilus* structure, all four subunits adopt a similar arrangement, generating a tetramer in which each MD lies adjacent to the NTD core. The cryo-EM structure of DnaB reported here, however, shows only two subunits adopt this compact NTD–MD arrangement [35]. The presence of only two resolved MDs can be rationalized by the relative positions of the interdomain linkers (Supplementary Fig. S6B and C). In chains B and C, residues 280–289 of the MD to CTD linker align antiparallel to residues 166–170 of chains D and A, respectively, generating two reciprocal linker pairs between chains A and C, and between chains B and D (Supplementary Fig. S6B). This arrangement prevents the NTD–MD linkers of chains A and D from wrapping around the NTD core. Instead, these linkers project away from the NTDs, displacing their MDs from the core region (Fig. 4B, and Supplementary Fig. S6B and C). As a result, only the MDs of chains B and C remain adjacent to the NTD core, forming the wing-like arrangement observed in the tetramer (Fig. 4A and B).

Despite the difference in symmetry between the two structures (Supplementary Fig. S6D and E), their domain level organization is broadly similar. The NTDs superpose with a root mean square deviation (RMSD) of 1.5 Å, while the MDs superpose with an RMSD of 1.6 Å. The NTD core adopts a dimer of dimers arrangement, like that observed in *G. stearothermophilus* DnaB. The dimer interfaces formed by chains A and B, and by chains C and D, each bury ∼3500 Å². These interfaces include contacts between the two NTDs within the core, together with contributions from the NTD–MD linker that wraps around the NTD core. In contrast, the interfaces between the two dimers are less similar, with the chains A and D interface burying 1735 Å², whereas the chains B and C interface buries 1367 Å². This difference arises mainly from helix α2 (Supplementary Fig. S6I). In chains A and D, α2 is extended and contributes to a larger buried interface area. In contrast, the corresponding helix is shortened in chains B and C. As a result, residue W59, which lies within α2 in chains A and D, is repositioned into the α2 to α3 loop in chains B and C. In this conformation the tryptophan is located closer to the MD linker of chain A, where it forms part of a hydrophobic cluster (Supplementary Fig. S6I).

To generate a complete structural model of DnaB, regions not resolved in the cryo-EM map were modelled using AF3 predictions for the individual chains (Supplementary Fig. S7) and incorporated into the experimental structure to produce a near complete tetrameric model of DnaB (Fig. 4C and Supplementary Fig. S7E–G). In this hybrid model, chains B and C retain the compact conformation observed in the cryo-EM structure, with wrapped linkers positioning the CTDs and CTTs close to the NTD core (Fig. 4C and D). By contrast, chains A and D adopt a more extended conformation, with outward projecting NTD to MD linkers placing MDs farther from the oligomerization core (Fig. 4C and D). The tetramer therefore adopts a two chain compact, two chain extended architecture that differs from both the fully symmetric *G. stearothermophilus* DnaB crystal structure and the full AF3 model of *B. subtilis* DnaB (Supplementary Fig. S6D–H).

The critical DnaB residues identified in the genetic screen (Figs 2 and 3) were mapped onto the hybrid structure (Fig. 4E) and topology diagram (Supplementary Fig. S8), revealing three distinct clusters located in the NTD (violet), CTD (yellow), and CTT (red) (Fig. 4E). The characterization of each cluster is described below.

### Identified DnaB residues are not required for self-interaction

The DnaB^NTD^ is responsible for tetramer assembly [21, 30, 35]. Although the critical DnaB residues identified in the NTD are located on the surface of the tetrameric core, we nonetheless started our analysis investigating DnaB self-interaction. Here each DnaB variant was tested against itself using the B2H assay. The results showed that all DnaB variants tested retained the ability to self-interact (Supplementary Fig. S9), indicating that loss of function was unlikely due to a defect in homo-oligomerization.

### DnaB^NTD^ residues mediate interaction with DnaA domain I

**Figure 5.**
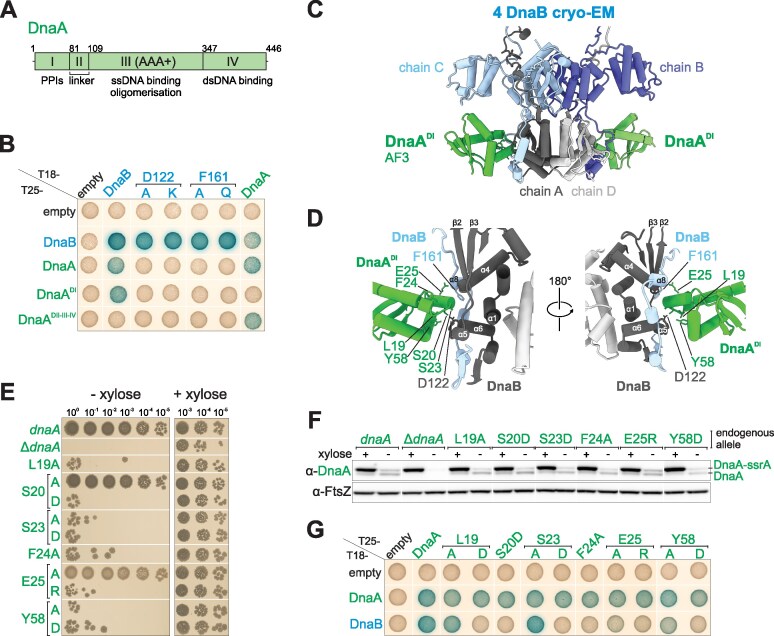
Identification and characterization of the DnaB–DnaA PPI. (**A**) Domain structure of *B. subtilis* DnaA. (**B**) B2H assay between DnaB variants and DnaA (full length or with domains truncated). Plate with X-gal at 0.008% (w/v) scanned at 48 h. (**C**) Structural prediction obtained using AF3 showing two individual molecules of DnaA domain I (DnaA^DI^, green) interacting with two available protomers of the tetrameric DnaB cryo-EM model. (**D**) Sections of the model shown in panel (C), centred on DnaA residues L19, S20, S23, F24, E25, Y58, and critical DnaB residues D122 and F161. (**E**) Spot titre assay showing growth of strains harbouring DnaA amino acid substitutions after 24 h. Xylose (0.5% w/v) was used to induce expression of an ectopic copy of *dnaA–ssrA*. (**F**) Immunoblot of DnaA variants following depletion of DnaA–ssrA for 90 min. The tubulin homolog FtsZ was used as a standard. Strains are the same as those in panel (E). (**G**) B2H assay between DnaA variants and DnaB. Plate with X-gal at 0.008% (w/v) scanned at 48 h.

Previous two-hybrid analysis detected a positive interaction between DnaB and DnaA, but specific residues involved in this PPI had not been identified [32]. DnaA is a multifunctional enzyme composed of four distinct domains that act in concert during initiation of DNA replication (Figs 1A and 5A) [71]. DnaA domain I (DnaA^DI^) is involved in PPIs [32, 33, 72] and domain II (DnaA^DII^) acts as a flexible linker. Domain III (DnaA^DIII^) is composed of an AAA+ motif that can assemble into an ATP-dependent right-handed helical oligomer [9, 73, 74] and also contains a ssDNA-binding site that specifically recognizes the trinucleotide DnaA-trio [8, 10, 75, 76]. Domain IV (DnaA^DIV^) contains a double-stranded DNA-binding motif that recognizes DnaA-box sequences [10, 77–79]. Because DnaA^DI^ is known to interact with the replication initiation factor DnaD and the sporulation regulator SirA [32, 33, 72], we hypothesized that DnaA^DI^ was the point of contact with DnaB.

B2H assays were performed using either wild-type DnaA or domain truncations (DnaA^DII-III-IV^ and DnaA^DI^). The results showed that DnaA^DI^ was necessary and sufficient to bind DnaB (Fig. 5B). Next, DnaB variants were screened by B2H for loss of interaction with DnaA. It was found that substitutions of D122 and F161 in DnaB inhibited the interaction with both DnaA and DnaA^DI^, suggesting that this cluster was involved in the PPI (Fig. 5B). All replication initiation proteins/fragments used for B2H showed at least one positive interaction, indicating that these fusions were being functionally expressed in *E. coli* (Fig. 5B).

The DnaB cryo-EM data and AF3 were used to probe the interaction between DnaA^DI^ and DnaB. AF3 predicts that DnaA^DI^ interacts with DnaB at a site containing both DnaB residues D122 and F161 (note that these residue pairs belong to distinct polypeptide chains, Fig. 5C and D, and Supplementary Fig. S10). Examination of the DnaA^DI^ interface suggests that DnaB^D122^ is proximal to DnaA residues L19, S20, S23, and Y58, while DnaB^F161^ is proximal to DnaA residues F24 and E25 (Fig. 5D).

To interrogate the AF3 structural prediction, mutations were constructed in the endogenous *dnaA* to alter the amino acids located at the proposed PPI (Supplementary Fig. S11). To allow mutagenesis of essential *dnaA* residues, an ectopic inducible *dnaA–ssrA* cassette was present in these strains (Supplementary Fig. S11). Spot titre analysis showed that at each position tested at least one DnaA substitution caused a severe growth defect (Fig. 5E). Immunoblots confirmed that the DnaA variants are stably expressed following depletion of DnaA–ssrA (Fig. 5F and Supplementary Fig. S12), indicating that these residues are critical for DnaA activity.

To assess the function of the critical DnaA^DI^ variants, PPIs with DnaB were investigated using B2H assay (Fig. 5G). At each position tested, at least one DnaA variant disrupted the interaction with DnaB. All DnaA^DI^ variants showed a positive interaction with wild-type DnaA, indicating that these fusion proteins were being functionally expressed in *E. coli* (Fig. 5G).

Notably, nuclear magnetic resonance using purified protein domains showed that DnaA residues S20 and S23 exhibited chemical-shift perturbations upon addition of DnaD, consistent with these side chains forming part of the PPI [33]. Indeed, B2H data suggests that DnaA residues involved in the DnaB PPI may also participate in the interaction with DnaD (Supplementary Fig. S13). Importantly, it appears unlikely that a single DnaA^DI^ could simultaneously bind DnaB and DnaD. Taken together, the data support the proposed structural model for DnaB^NTD^ binding DnaA^DI^ and is consistent with the established role of DnaA^DI^ as a PPI hub.

### DnaB^CTD^ residues mediate interaction with the DnaD C-terminal tail

**Figure 6.**
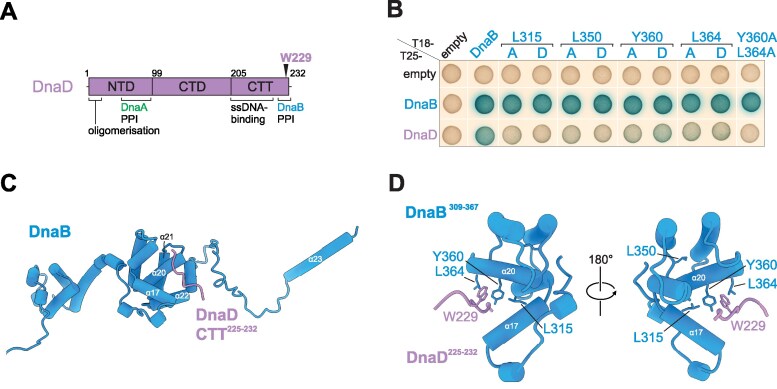
Identification and characterization of the DnaB–DnaD PPI. (**A**) Domain structure of *B. subtilis* DnaD. (**B**) B2H assay between DnaB variants and DnaD. Plate with 0.008% (w/v) X-gal scanned at 48 h. (**C**) Structural prediction obtained using AF3 showing one chain of DnaB (blue) and the distal end of DnaD^CTT^ (residues 225–232, pink). (**D**) Structural prediction obtained using AF3 showing sections of DnaB^CTD^ and DnaD^CTT^, highlighting critical residues DnaB L315, L350, Y360 and L364, and DnaD W229.

Previous alanine-scanning analyses of *B. subtilis* DnaD identified critical residues within the NTD (DnaD^NTD^) that mediate an interaction with DnaA, while the DnaD C-terminal tail (DnaD^CTT^) contains a ssDNA-binding motif and mediates an essential interaction with DnaB (Fig. 6A) [32, 34]. Two-hybrid analysis has shown that either deleting the last eight amino acids from the DnaD^CTT^ (residues 225–232) or substituting tryptophan at position 229 with alanine abolishes/decreases the interaction with DnaB, respectively [32]. Additionally, two-hybrid analysis has detected interactions between DnaD^NTD^ and both DnaB^NTD^ and DnaB^CTD–CTT^ [27].

DnaB variants were screened by B2H assay and it was found that substitutions of residues L315, L350, Y360, and L364 perturb the interaction with DnaD (Fig. 6B). All DnaB variants used for this B2H assay showed a positive interaction with wild-type DnaB, indicating that these fusions were being functionally expressed in *E. coli* (Fig. 6B).

AF3 was used to predict the interaction between DnaB and the distal end of the DnaD^CTT^ (residues 225–232) (Fig. 6C and D, and Supplementary Fig. S14A). Modelling suggested that this region of the DnaD^CTT^ engages DnaB within a groove formed by two α-helices harbouring three critical DnaB residues (L315, Y360, L364 within ⍺17 and α20 ) (Fig. 6D). The essential DnaD^W229^ [32] can be seen wedged between these two DnaB α-helices (Fig. 6D). DnaB residue L350 abuts α20, suggesting that amino acid substitutions at this position may alter DnaD binding indirectly (Fig. 6D). Together, the data are consistent with the proposed structural model for the DnaB^CTD^ binding to the distal end of a DnaD^CTT^.

### DnaB^CTT^ residues mediate interaction with the DnaI CTD

**Figure 7.**
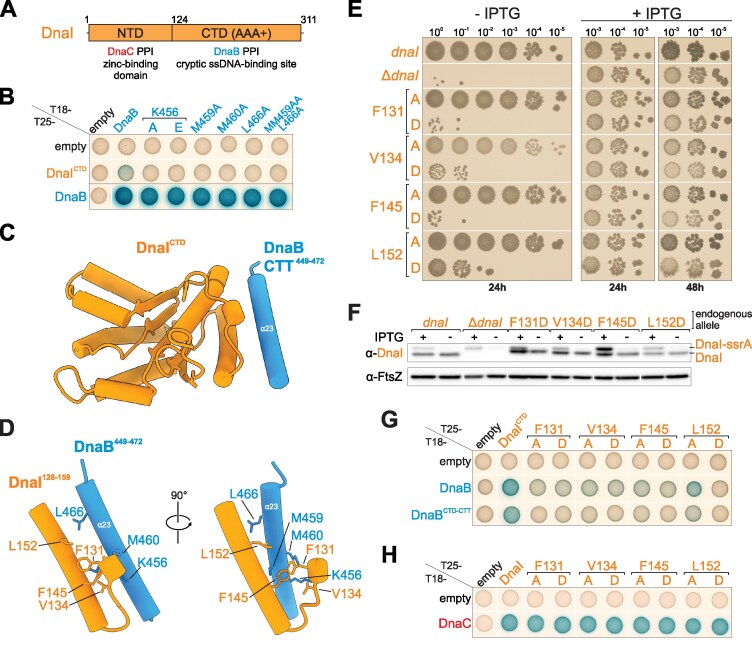
Identification and characterization of the DnaB–DnaI PPI. (**A**) Domain structure of *B. subtilis* DnaI. (**B**) B2H assay between DnaB variants and DnaI^CTD^. Plate with X-gal at 0.016% (w/v) scanned at 47 h (bottom) and 71 h (top). (**C**) Structural prediction obtained using AF3 showing one DnaB^CTT^ (residues 449–472 shown, blue) and the DnaI^CTD^ (orange). (**D**) Section of the model shown in panel C, centred on DnaB critical residues K456, M459, M460, and L466, and DnaI residues F131, V134, F145, and L152. (**E**) Spot titre assay showing growth of strains harbouring DnaI amino acid substitutions after 24 or 48 h. IPTG (0.1 mM) was used to induce expression of an ectopic copy of *dnaI–ssrA*. (**F**) Immunoblot of DnaI variants following depletion of DnaI–ssrA for 90 min. The tubulin homolog FtsZ was used as a standard. Strains are the same as those in panel (E). (**G**) B2H assay between DnaI^CTD^ variants and DnaB (full length or DnaB^CTD–CTT^). Plate with X-gal at 0.016% (w/v), scanned at 71 h. (**H**) B2H assay between DnaI variants and DnaC. Plate with X-gal at 0.008% (w/v), scanned at 24 h.

The helicase loader DnaI can be structurally divided into an NTD (DnaI^NTD^) and a CTD (DnaI^CTD^) (Fig. 7A). The DnaI^NTD^ contains a DnaC helicase-interacting domain (zinc-binding domain) [25, 27, 80, 81], while the DnaI^CTD^ consists of an AAA+ domain and harbours a cryptic ssDNA-binding site [11, 82]. It has been reported that CTDs of DnaB (CTD–CTT, residues 303–472) interact with the DnaI^CTD^ [27]. As the role of critical DnaB^CTT^ residues had not yet been assigned (Figs 2D–G, 3, and 4E), we hypothesized that this cluster constitutes the primary interaction site for DnaI.

DnaB variants were screened by B2H assay, and it was found that substitutions of residues K456, M459, M460, and L466 decreased interaction with the DnaI^CTD^ (Fig. 7B). All DnaB variants used for B2H showed a positive interaction with wild-type DnaB, indicating that these fusions were being functionally expressed in *E. coli* (Fig. 7B).

AF3 was used to predict the interaction between the DnaB^CTT^ and the DnaI^CTD^ (Fig. 7C and D, and Supplementary Fig. S14B). The results suggested that DnaB^CTT^ residues K456, M459, M460, and L466 form an interface with DnaI^CTD^ residues F131, V134, F145, and L152, positioning several hydrophobic residues towards one another (Fig. 7D).

To interrogate this model, mutations were introduced into the endogenous *dnaI* allele to alter amino acids located at the proposed DnaB–DnaI interface. To allow mutagenesis of essential *dnaI* residues, an ectopic inducible *dnaI–ssrA* complementation system was present in these strains (Supplementary Fig. S15). Spot titre analysis showed that while alanine substitutions were tolerated at all positions, substitution with the negatively charged aspartic acid caused severe growth defects (Fig. 7E), consistent with formation of a hydrophobic PPI between DnaB and DnaI. Immunoblots confirmed that the DnaI variants are stably expressed following depletion of DnaI–ssrA, indicating that these residues are critical for DnaI activity (Fig. 7F and Supplementary Fig. S16).

To assess the function of DnaI residues, the interaction of DnaI^CTD^ variants with DnaB was tested using the B2H assay. At each position, there was at least one DnaI^CTD^ variant that diminished interaction with DnaB (Fig. 7G). To assess whether substitutions in DnaI compromised protein expression, each mutation was introduced into full-length DnaI and interactions were tested against the replicative helicase (DnaC). All DnaI variants showed a positive interaction with DnaC, indicating that these amino acid substitutions are unlikely to compromise protein stability in *E. coli* (Fig. 7H). Taken together with previous results [27], the data support the proposed model for the DnaB^CTT^ binding to the DnaI^CTD^.

### Critical DnaB PPIs are predicted to be conserved in human pathogens

To assess conservation of DnaB interaction interfaces, sequence alignments of DnaB, DnaA, DnaD, and DnaI homologs from Firmicute pathogens were analysed (Supplementary Fig. S17, and Supplementary Tables S11–S15) [60]. Alignments indicate that the DnaB–DnaA and DnaB–DnaD PPIs are highly conserved (Supplementary Fig. S17A and B). In contrast, the DnaB–DnaI PPI appears less conserved (Supplementary Fig. S17C), although the prevalence of nonpolar residues is consistent with a hydrophobic interface (Fig. 7C and D). These results suggest that DnaB PPIs are conserved in Firmicutes pathogens using the DnaB helicase loading system.

## Discussion

### DnaB is an interaction hub for DNA replication initiation proteins in *B. subtilis*

Despite the universal importance of DNA replication initiation for faithful genome duplication, crucial aspects of this process remain poorly understood in bacteria. Particularly unclear are the precise, physiologically relevant interactions between initiation proteins that enable productive replicative helicase loading at the origin prior to the onset of bidirectional DNA synthesis.

Here we have investigated the helicase loading mechanism used by several Firmicutes, involving an AAA+ helicase loader (DnaI) and the accessory proteins DnaD and DnaB. A genetic screen identified residues in DnaB necessary for its function during DNA replication initiation (Figs 2 and 3). B2H analysis and biomolecular structural predictions indicate that most of these critical residues are interaction sites for other essential DNA replication initiation proteins (DnaA, DnaD, DnaI, Figs 5–7). Protein alignments suggest that these PPIs are conserved (Supplementary Fig. S17).

A previous B2H analysis found that the DnaB^CTD–CTT^ interacts with the DnaC^CTD^ [27]. While our B2H analyses also detected an interaction between DnaB and DnaC, no single DnaB alanine variant was found to disrupt this interface (Fig. 1B). This suggests that either the DnaB–DnaC PPI is extensive, such that no single alanine substitution causes significant disruption, or that this interaction may not be critical *in vivo*.

The function(s) of DnaB residues D337, Y374, and R384 remain unknown (Figs 2E and F, and 3). B2H analysis showed that substitutions at these positions did not diminish interactions with DnaA, DnaD, DnaI, or DnaC (Supplementary Fig. S18A). Interestingly, these residues cluster in and around a previously identified ^374^YxxxIxxxW^382^ motif in the DnaB^CTD^ (Supplementary Fig. S18B) [22] and alanine variants all displayed a cold-sensitive phenotype (Supplementary Fig. S18C). DNA binding assays and size-exclusion chromatography of Y374A, I378A, and W382A variants support a role of these residues in higher-order oligomerization [31]. Further investigation will be necessary to clarify the precise role of these residues.

### Model of DnaB interacting with DnaA, DnaD, and DnaI

**Figure 8.**
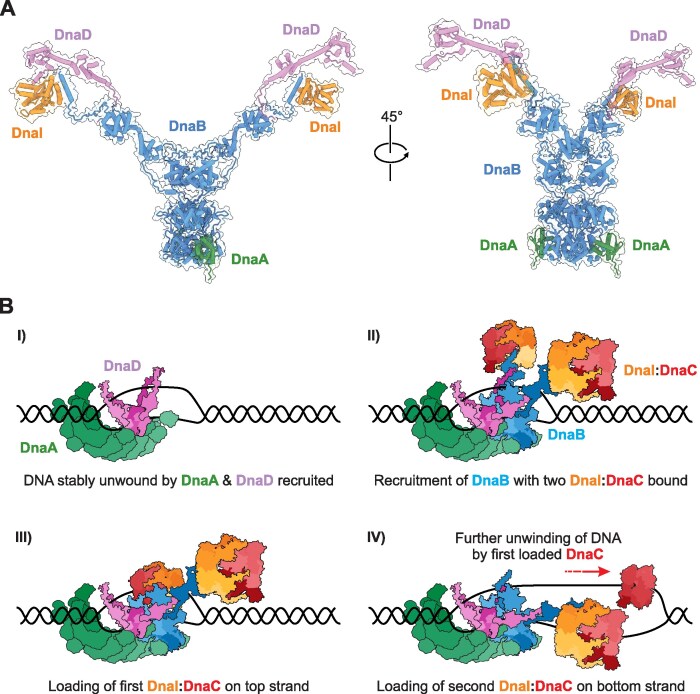
Models for *B. subtilis* DnaB activity during DNA replication initiation. (**A**) The DnaB hybrid model (full-length tetramer) shown with monomeric binding partners DnaA (domain I extending into domain II), DnaD (full-length) and DnaI (CTD). (**B**) Proposed model for *B. subtilis* DNA replication initiation. (I) DnaA unwinds *oriC* and recruits DnaD; (II) the helicase loader complex of DnaB:DnaI:DnaC is recruited to *oriC* by DnaA and DnaD; (III) one DnaI:DnaC complex is first deposited onto the strand opposite DnaA; (IV) the initial replicative helicase moves away from *oriC* and enlarges the open complex, providing space for the second DnaI:DnaC complex to be loaded onto the opposite strand.

Single particle cryo-EM of purified DnaB revealed an unexpected two-fold symmetry generated by the tetrameric oligomer. Taken together with genetic results, two-hybrid analysis, and AF3 structural predictions, the data allow us to propose a PPI model for this class of bacterial helicase loaders (Fig. 8A). Important features of the proposed model are highlighted below.

The cryo-EM model shows that two MDs of the DnaB tetramer contact the NTD core, while the other two do not (Fig. 4B and C). Such positioning of the MDs against the NTD occludes DnaA^DI^ binding sites on two of the chains. Ultimately in this conformation, up to two DnaA protomers could interact with one DnaB tetramer at the identified DnaB^NTD^ interface.

The structural model also suggests that MD–CTD–CTT of two DnaB protomers point away from the tetrameric core, and we propose that this extended conformation may accommodate binding of two helicase loading complexes (DnaI_6_:DnaC_6_) (Figs 4C and D, and 8). Inherent flexibility between the MD–CTD and the CTD–CTT likely allows conformational freedom during loading (Fig. 4D). Therefore, we propose that a single DnaB tetramer can load both helicases at the replication origin, with two extended DnaB protomers directing loading DnaI:DnaC complexes on opposite strands (Fig. 8B).

AF3 predicts that the CTD and CTT of DnaB interact (Fig. 4D). If so, this self-interaction would occlude binding sites for both DnaD and DnaI (Fig. 4E). Based on the observed dependency of replication initiation protein recruitment to *oriC* (DnaA → DnaD → DnaB → DnaI) [24], binding of DnaD to DnaB may inhibit the CTD–CTT self-interaction and liberate the CTT, making it available to bind DnaI. Likewise, domain I of DnaA appears to use an overlapping interaction surface to contact DnaD and DnaB (Supplementary Fig. S13), suggesting that these PPIs may be mutually exclusive. Determining the dynamics of DnaB PPIs will be necessary to deconvolute these early stages of helicase loading.

The size of the open complex at *B. subtilis oriC* has been measured to be ∼40 unpaired bases, extending from DnaA-trios towards an adjacent AT-rich motif [62, 83]. Considering the length of ssDNA within a helicase loader complex (26 nucleotides) [16], potential steric clashes with DnaA and DnaD [10, 34], and requirement for two helicase loading events, we hypothesize that replicative helicases are loaded sequentially (Fig. 8B). We favour the model that a first helicase is loaded on the strand containing the DRE [34], as this helicase would translocate in the 5′→3′ direction and enlarge the open complex, providing space for a second helicase to be loaded on the opposite strand. Interestingly, in this model the second helicase would translocate towards the DnaA oligomer, potentially removing DnaA to inhibit further DNA replication initiation, as previously suggested for *E. coli* [84, 85].

## Supplementary Material

gkag630_Supplemental_Files

## Data Availability

All plasmids and strains are available upon request. The experimental cryo-EM map and corresponding atomic model have been deposited in the Electron Microscopy Data Bank (EMDB) and the Protein Data Bank (PDB), respectively, under accession codes EMDB EMD-57240 and PDB DOI https://doi.org/10.2210/pdb29km/pdb. Structural models have been deposited in the ModelArchive (https://modelarchive.org/) repository: DnaB tetramer (ma-k2gxa), DnaB–DnaA PPI (ma-nocqz), DnaB–DnaD PPI (ma-9osww), DnaB–DnaI PPI (ma-l1wpm), Hybrid cryoEM-AlphaFold3 PPI (ma-v3shm).
