# Supplementary material for: Bacillus subtilis DnaB forms multiple protein–protein interactions essential for DNA replication initiation
Source: Nucleic Acids Res. 2026 Jul 2;54(12):gkag630. doi: 10.1093/nar/gkag630 (PMC13326638; doi:10.1093/nar/gkag630)
Supplement: gkag630_Supplemental_Files [file gkag630_supplemental_files.zip › Guyet_SupplementalFigures_Legends_20260529_clean.pdf]

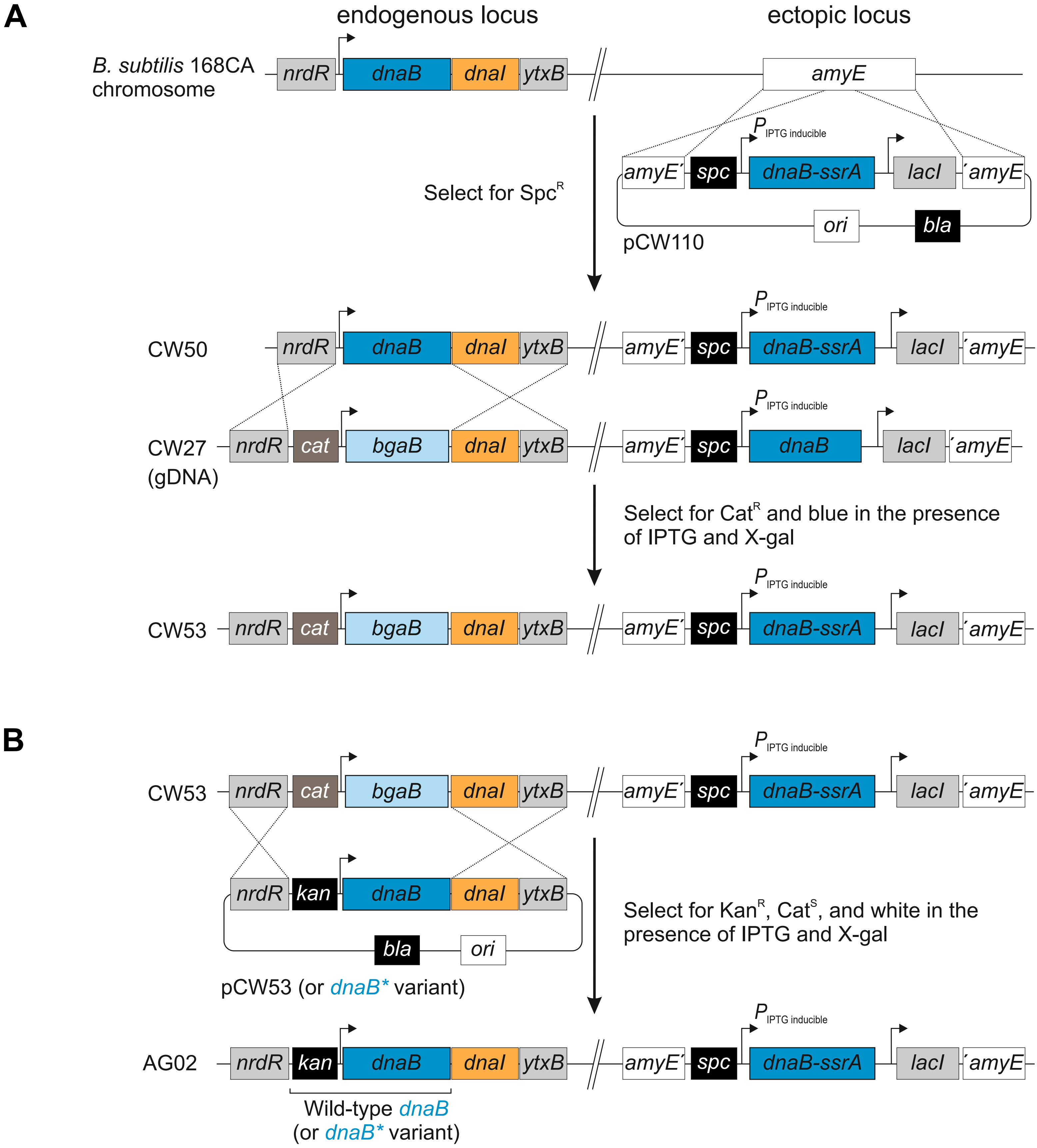

Supplementary Figure S1

Figure S1. Genetic complementation assay for functional analysis of *dnaB* variants in *B. subtilis*. (**A**) Construction of the recipient strain, CW53, for blue/white screening of DnaB variants in *B. subtilis.* An IPTG inducible *dnaB-ssrA* allele (ectopic) was inserted at the non-essential *amyE* locus by spectinomycin (Spc^R) selection, followed by replacement of the endogenous *dnaB* with *bgaB* (encoding a β-galactosidase, placed under the control of the constitutive P_{veg} promoter). The final strain, CW53, was isolated by selecting for a blue colony on medium supplemented with IPTG, X-gal, and chloramphenicol (Cat^R). (**B**) Schematics of the *dnaB* blue/white screening assay. CW53 was transformed with pCW53 carrying *dnaB* or a *dnaB** variant (derivative plasmid obtained by PCR site-directed mutagenesis) selected for kanamycin resistance (Kan^R), and screened for a white phenotype in the presence of IPTG and X-gal. White colonies were verified to be chloramphenicol sensitive (Cat^S) and Spc^R on IPTG-containing medium.

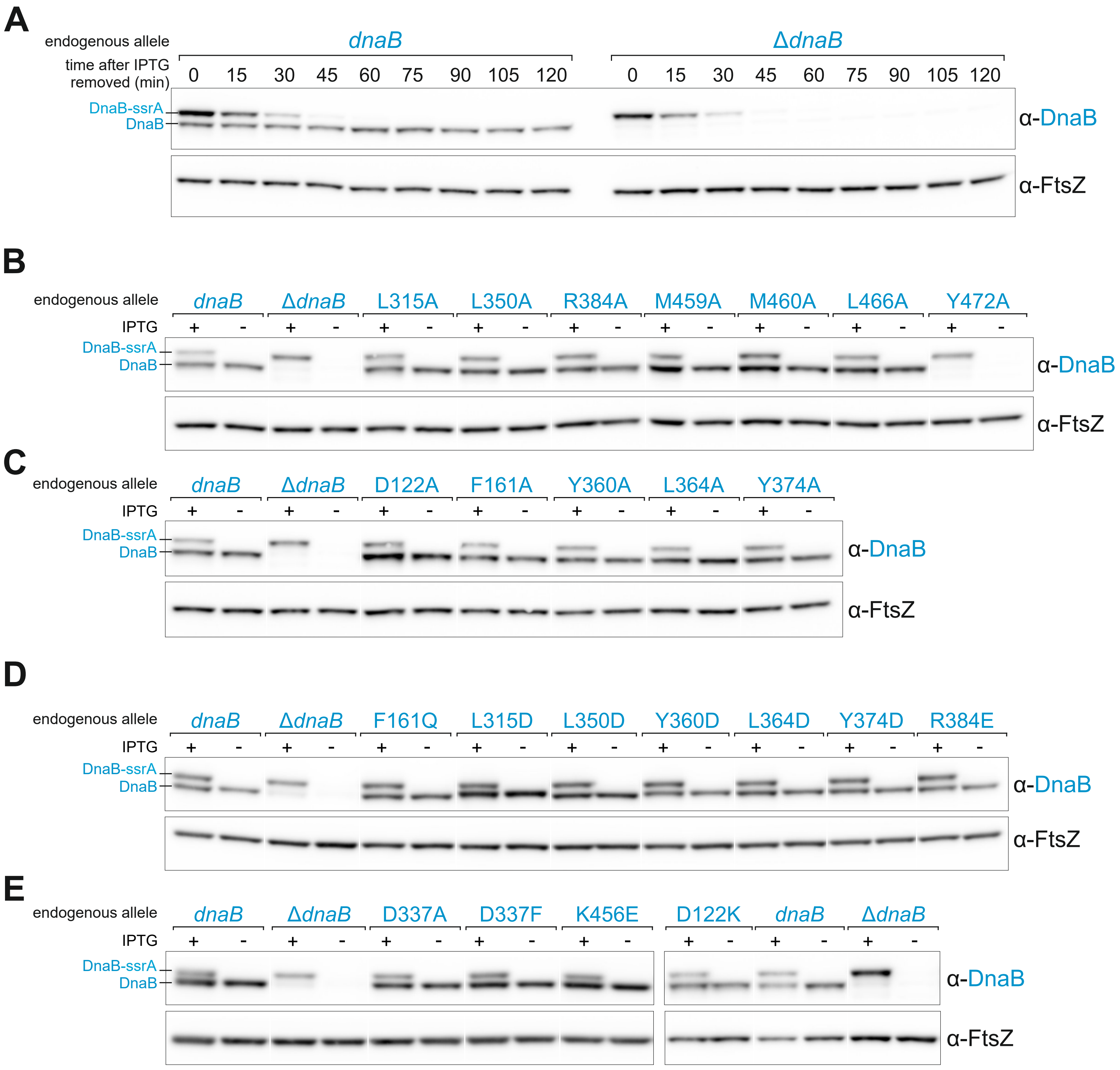

Figure S2. Depletion and expression of inducible DnaB-ssrA and DnaB variants in *B. subtilis*. (**A**) Immunoblot of wild-type and Δ*dnaB* strains following depletion of DnaB-ssrA. Exponential growing cultures containing IPTG were washed, resuspended in medium with or without IPTG (0.1 mM), and grown for an additional 90 min. Significant degradation of DnaB-ssrA was observed within 45 min. A faint, non-specific band with a slightly higher molecular weight than DnaB was also observed. The tubulin homolog FtsZ was used as a loading control. (**B-E**) Immunoblots showing *in vivo* expression of tested DnaB variants. Control strains and loading control are the same as in (A).

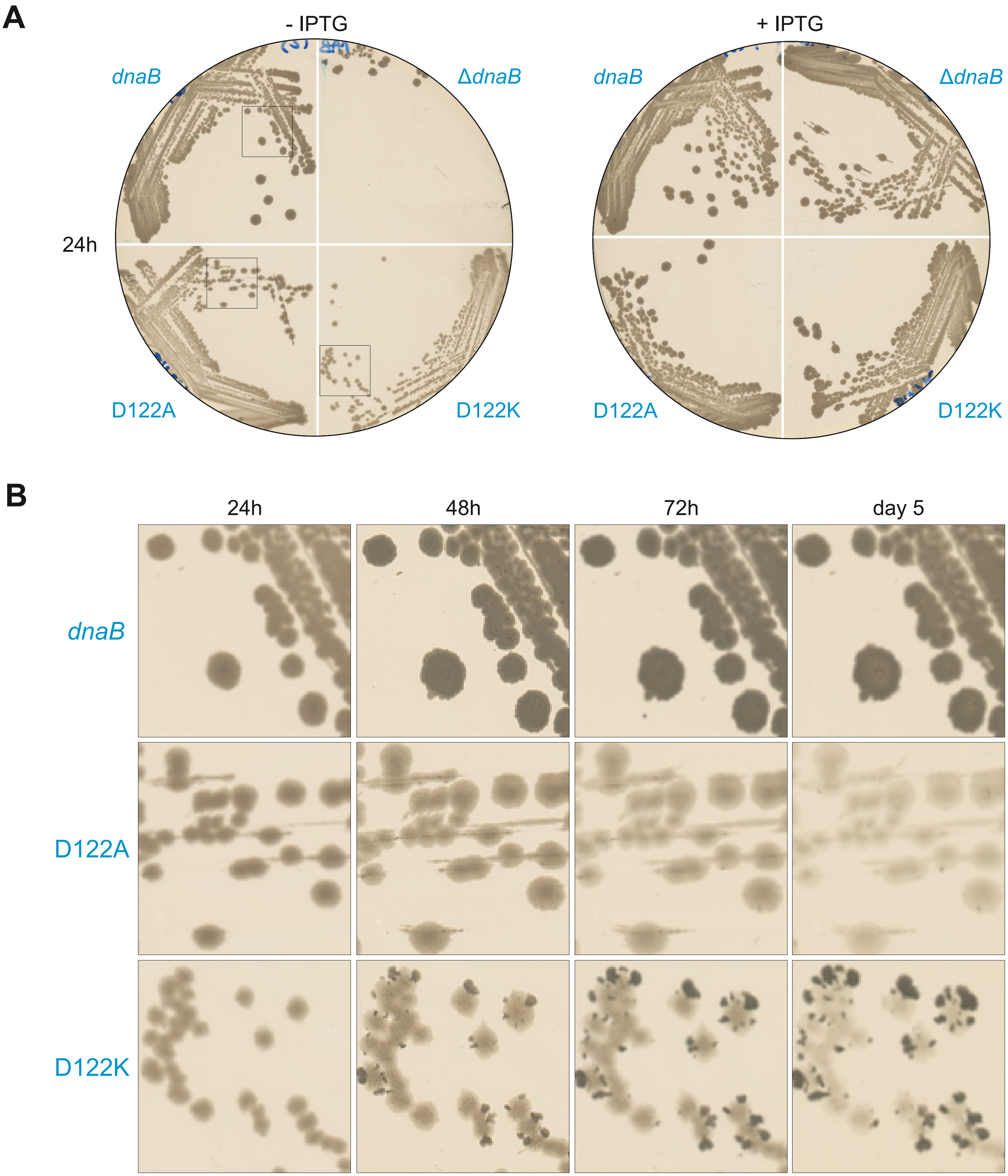

Figure S3. Defective phenotype of DnaB^{D122K} variant.

(**A**) Phenotypic defect associated with DnaB^{D122K} variant. Strains carrying either wild-type *dnaB*, Δ*dnaB,* or the D122A and D122K variants were streaked on PAB agar in the absence (-) or presence (+) of IPTG (0.1 mM) and incubated at 37 ºC. Plates were scanned at multiple time points over 5 days; representative scans at 24 h are shown. (**B**) Enlarged views of framed regions from the plate without IPTG, highlighting single colonies at different time points.

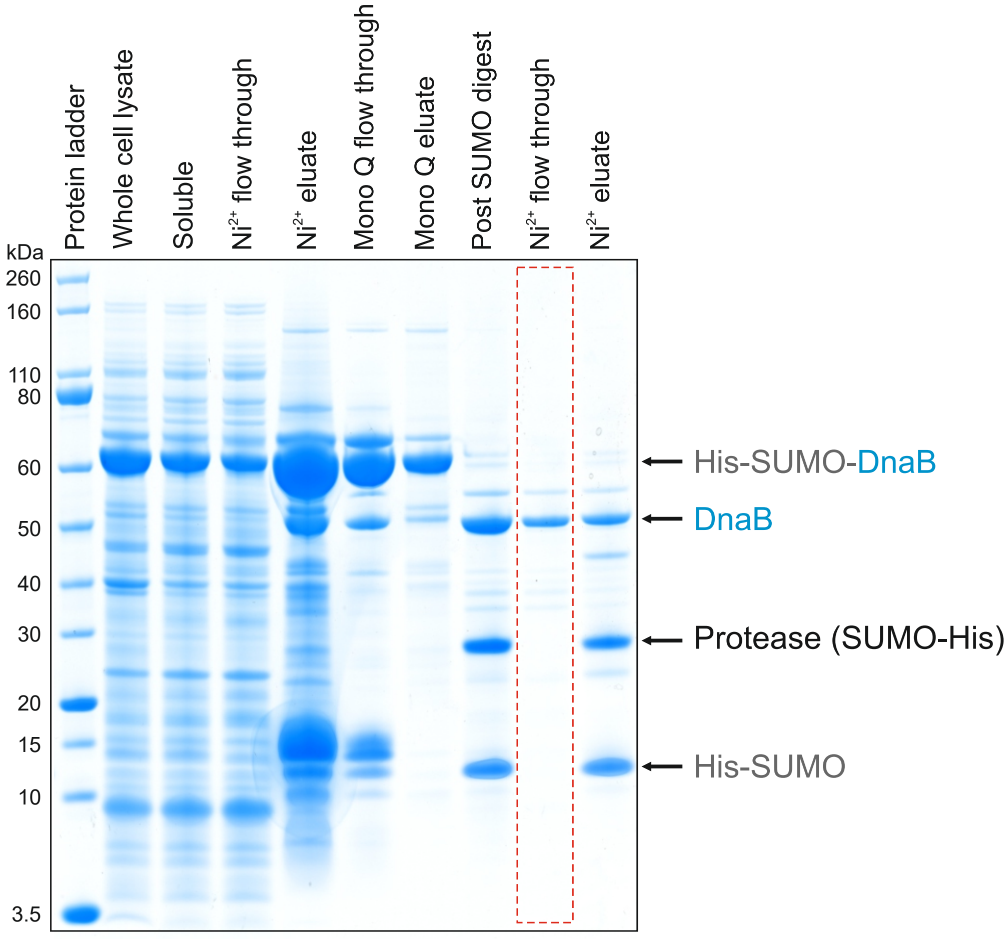

Figure S4. Purification of *B. subtilis* DnaB for cryo-EM

SDS-PAGE showing purification steps for DnaB. Soluble His-SUMO-DnaB was purified first using nickel affinity chromatography and second using ion exchange chromatography. The purified His-SUMO-DnaB was digested by SUMO-His protease, releasing the His- and SUMO-tag. The reaction was applied to a second nickel affinity column, and untagged DnaB was collected in the eluate. Purification steps are indicated above each lane, and the sample used for cryo-EM analysis is outlined (dashed red box).

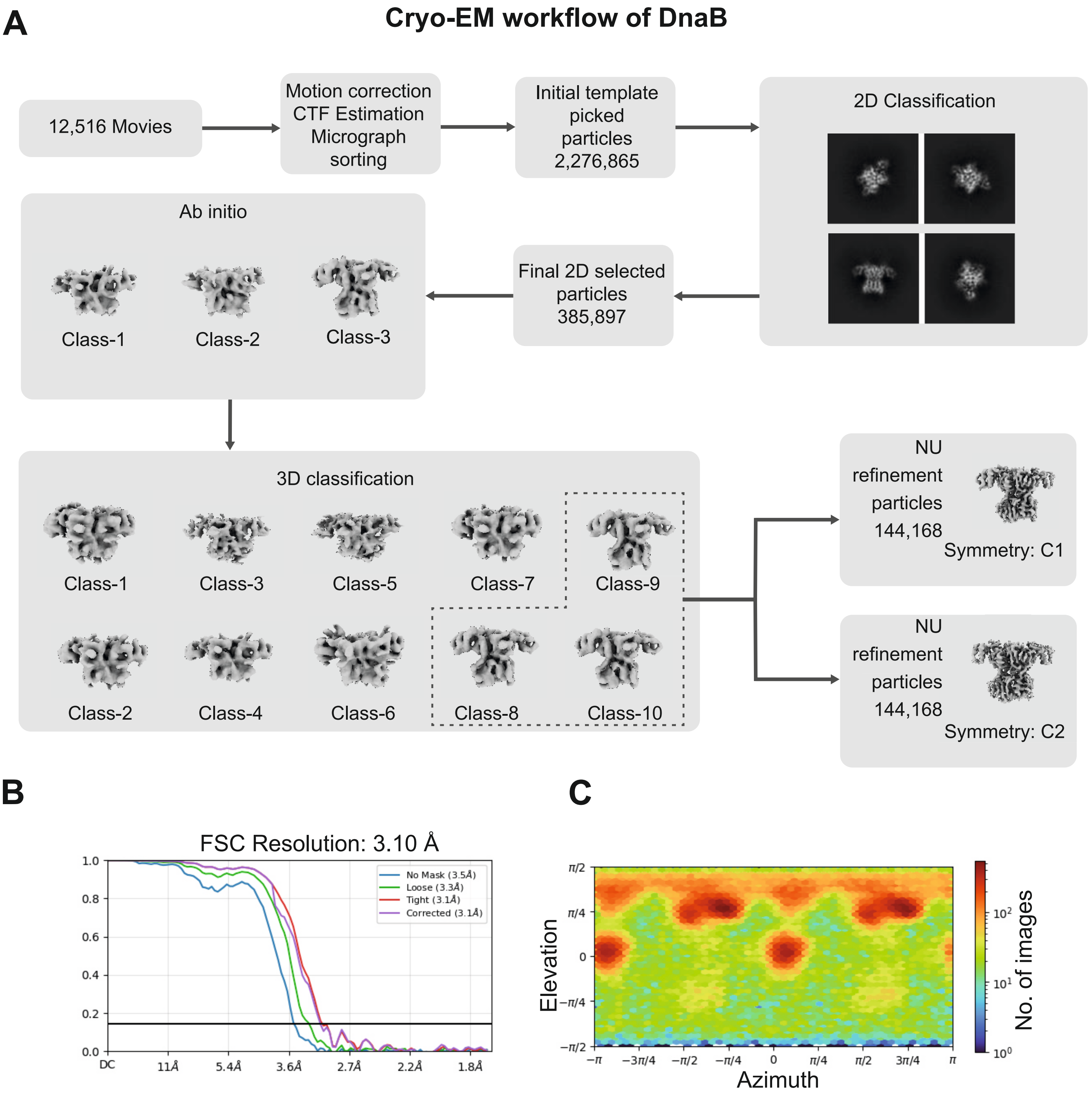

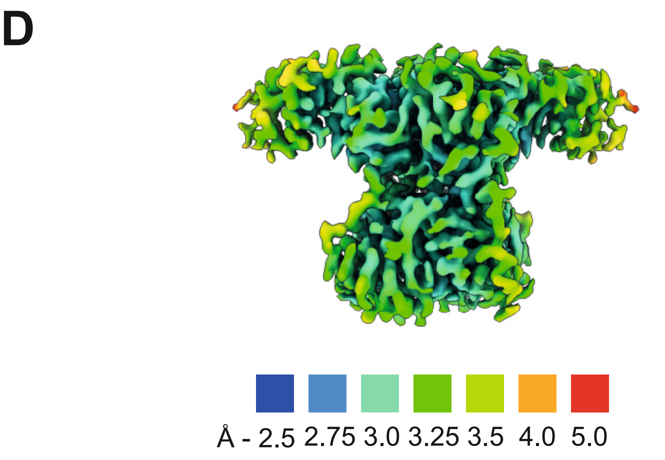

Supplementary Figure S5

Figure S5. Cryo-EM data processing of DnaB tetramer

(**A**) The single particle cryo-EM data processing workflow of the DnaB tetramer. (**B**) Fourier shell correlation (FSC) of the DnaB tetramer map showing an average map resolution of 3.1 Å. (**C**) Orientation distribution of particles contributing for the cryo-EM map of the DnaB tetramer. (**D**) The cryo-EM map coloured by local resolution contoured at 0.24 σ.

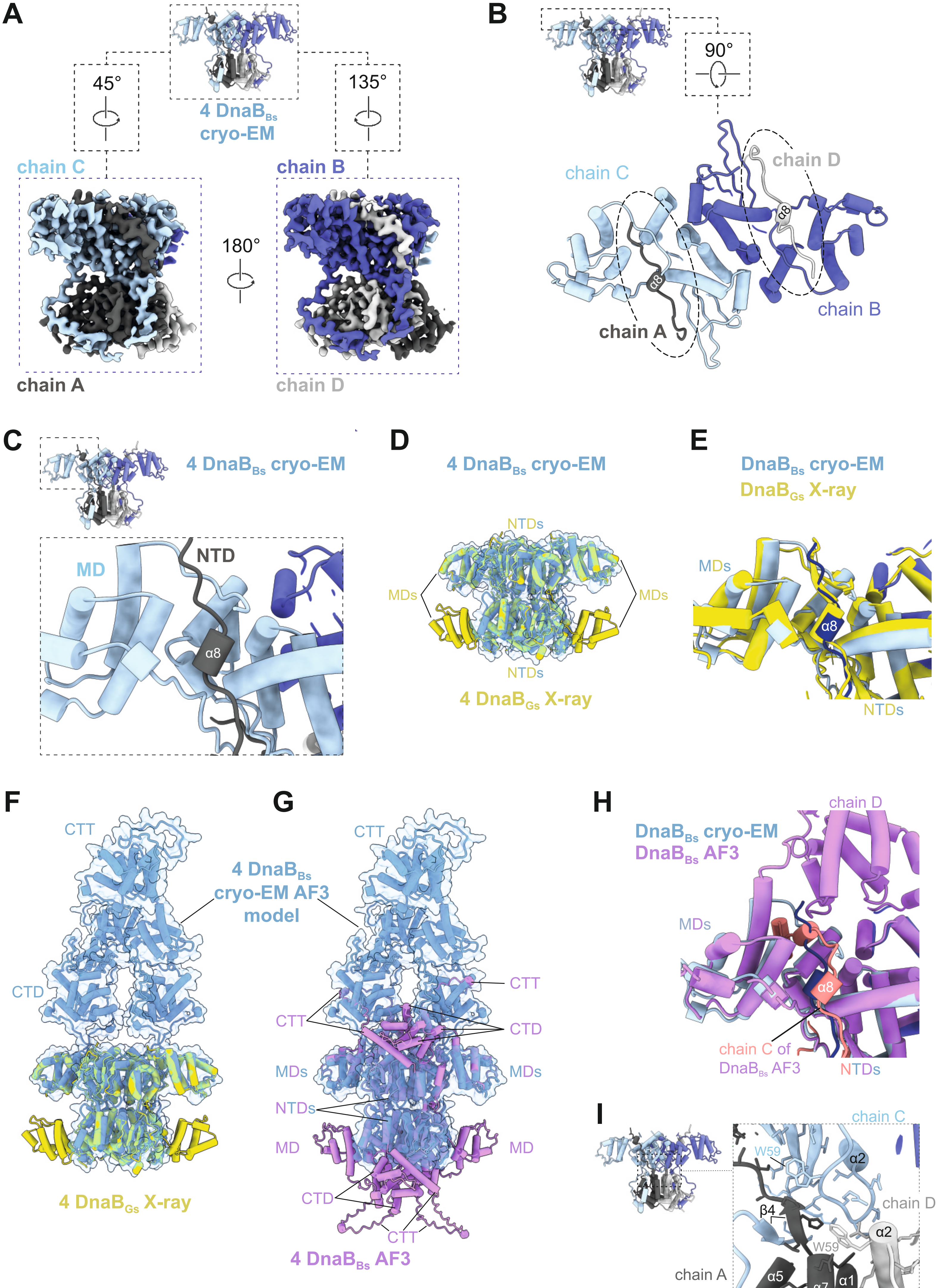

Supplementary Figure S6

Figure S6. Structural comparison of the DnaB tetramer

(**A**) Cryo-EM map of *B. subtilis* DnaB tetramer with chains individually coloured (at reduced scale) with the boxed region magnified. The clipped density map shows continuous density between the NTD and MD of chain C (light blue), wrapping around the NTD core of chain A (dark blue). The resolved end of chain A projects outwards from the NTD core (**B**) Cartoon representation of the cryo-EM DnaB tetramer coloured based on the individual chains, with the dashed region magnified. The short resolved NTD ends (linker residues 166-171) of chain A (dark blue) and chain D (cyan) extend away from the structure core and adopt an antiparallel arrangement to the resolved ends (linker residues 280-289) of chain C (light blue) and B (blue), respectively. (**C**) Same structure as in B, from a different viewpoint highlighting the resolved ends of chain A NTD (residues 166-171) and chain C MD (residues 280-289) showing antiparallel alignment of these linkers. Panels A to C illustrate the asymmetric protomer organisation within DnaB tetramer. (**D**) Structural alignment of DnaB models from *B. subtilis* (cryo-EM) in blue ($DnaB_{Bs}$) and *G. stearothermophilus* (X-ray crystallography) in yellow ($DnaB_{Gs}$). (**E**) Same as panel-C with the X-ray crystal structure of *G. stearothermophilus* (yellow) $DnaB_{Gs}$ aligned. (**F**) Structural alignment of DnaB models from *B. subtilis* (cryo-EM-AlphaFold hybrid) in blue and *G. stearothermophilus* X-ray crystallography (yellow). (**G**) Structural alignment of the *B. subtilis* DnaB models from cryo-EM-AlphaFold hybrid model (blue) and from AlphaFold 3 (purple). (**H**) Same as panel-C with the structure of AlphaFold3 (AF3) model (purple, with chain C in red) of *B. subtilis* DnaB aligned. (**I**) Cryo-EM map of *B. subilis* DnaB tetramer with chains individually coloured (at reduced scale) with the boxed region magnified. Close-up view of residue W59 in chain C highlighing its surrounding hydrophobic residues in the molecules with a shortened α2 helix, and comparison with W59 in chain D where α2 is extended.

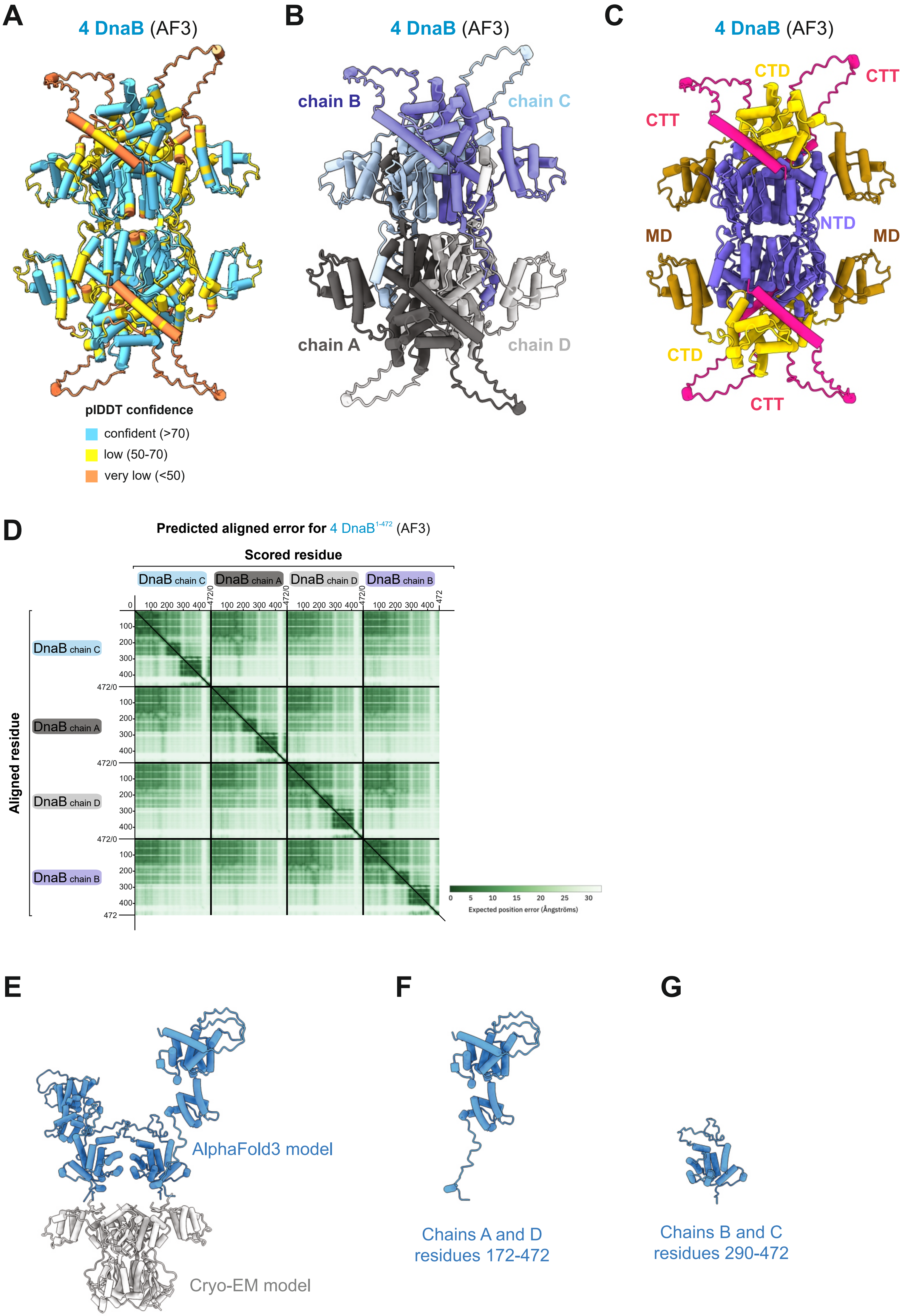

A
4 DnaB (AF3)
pIDDT confidence
confident (>70)
low (50-70)
very low (<50)
B
4 DnaB (AF3)
chain B
chain C
chain A
chain D
C
4 DnaB (AF3)
CTD
CTT
CTT
MD
NTD
MD
CTD
CTT
D
Predicted aligned error for 4 DnaB1-472 (AF3)
Scored residue
DnaB chain C
DnaB chain A
DnaB chain D
DnaB chain B
Aligned residue
Expected position error (Ångströms)
E
AlphaFold3 model
Cryo-EM model
F
Chains A and D
residues 172-472
G
Chains B and C
residues 290-472

Figure S7. Predicted alignment error for AlphaFold3 model of DnaB tetramer

(**A**) AlphaFold3 model for the DnaB tetramer was used to build the hybrid model shown in **Figures 4C-E**. The AlphaFold3 DnaB tetramer model is represented by predicted local Distance Difference Test (plDDT) with confident (in blue, plDDT > 70), low (in yellow, 70 > plDDT > 50) and very low (in orange, plDDT < 50) confidence. (**B**) AlphaFold3 DnaB tetramer chains are individually coloured. (**C**) AlphaFold3 DnaB tetramer coloured by domains, as defined in **Figure 2C**. (**D**) The predicted alignment error (PAE) for the DnaB tetramer model is provided with each chain indicated. (**E**) DnaB hybrid model generated by combining the cryo-EM model (grey) with AlphaFold3 derived regions (blue). (**F**) Chains A and D of the AlphaFold3 component of the DnaB hybrid model. The AlphaFold3 derived region corresponds to residues 172-472. (**G**) Chains B and C of the AlphaFold3 component of the DnaB hybrid model. The AlphaFold3 derived region corresponds to residues 290-472.

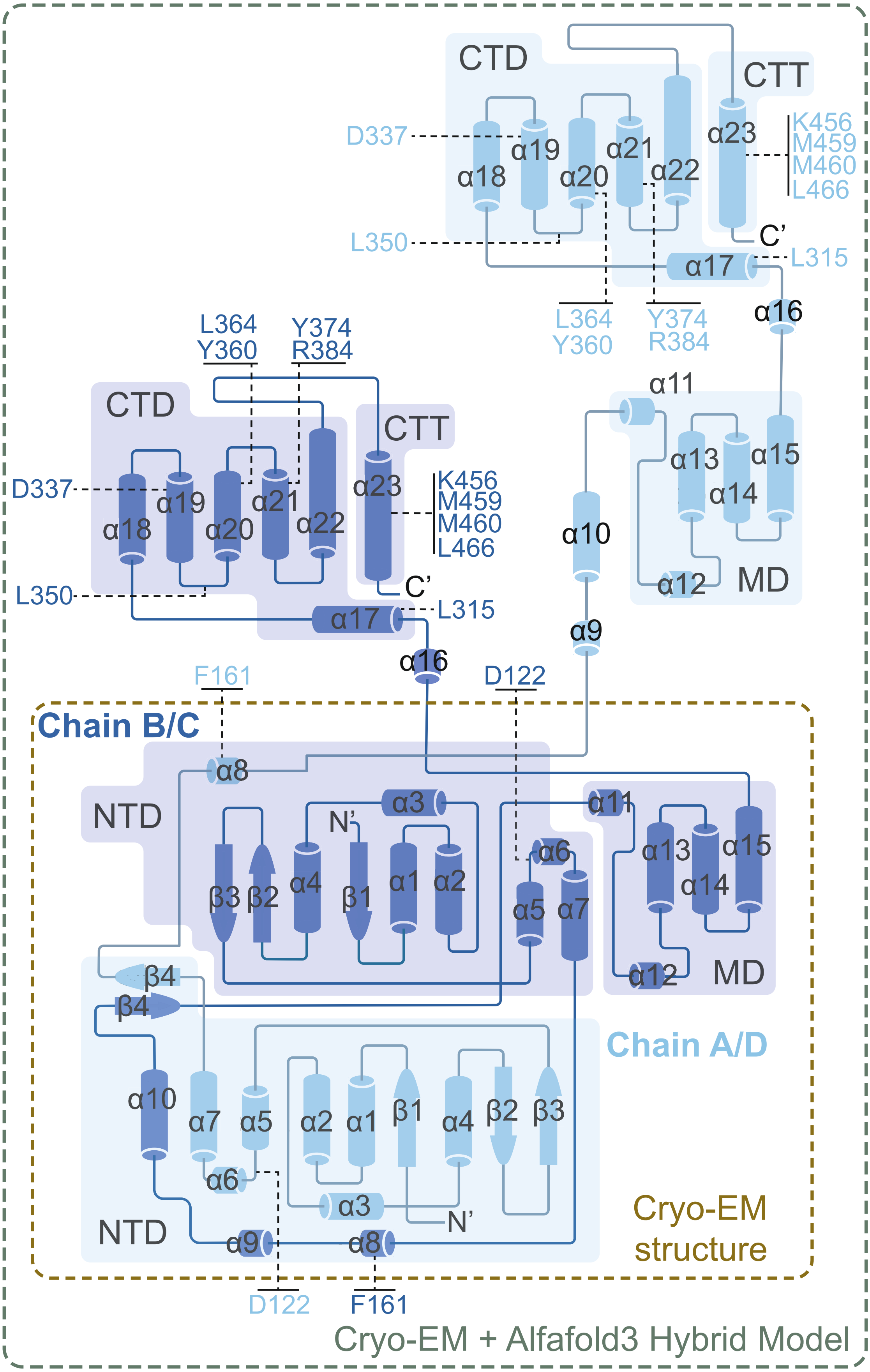

Figure S8. Topology diagram of DnaB

A topology diagram of *B. subtilis* DnaB chains B and D is presented with dashed lines indicating boundaries for the cryo-EM structure and for the cryo-EM-AlphaFold hybrid model. DnaB critical residues identified in this study are positioned within the topology.

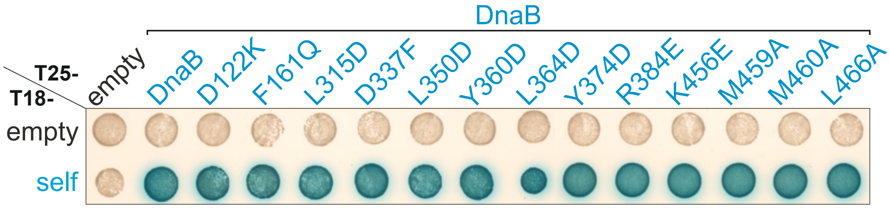

Figure S9. DnaB variants are capable of oligomerisation

B2H assay showing oligomerisation of DnaB variants. Each DnaB variant was fused to both T18 and T25 fragments to assess homologous interactions. 'Self' indicates the co-expression with the same DnaB variant indicated on the top x-axis. Plate with 0.008% (w/v) X-gal scanned at 48 h.

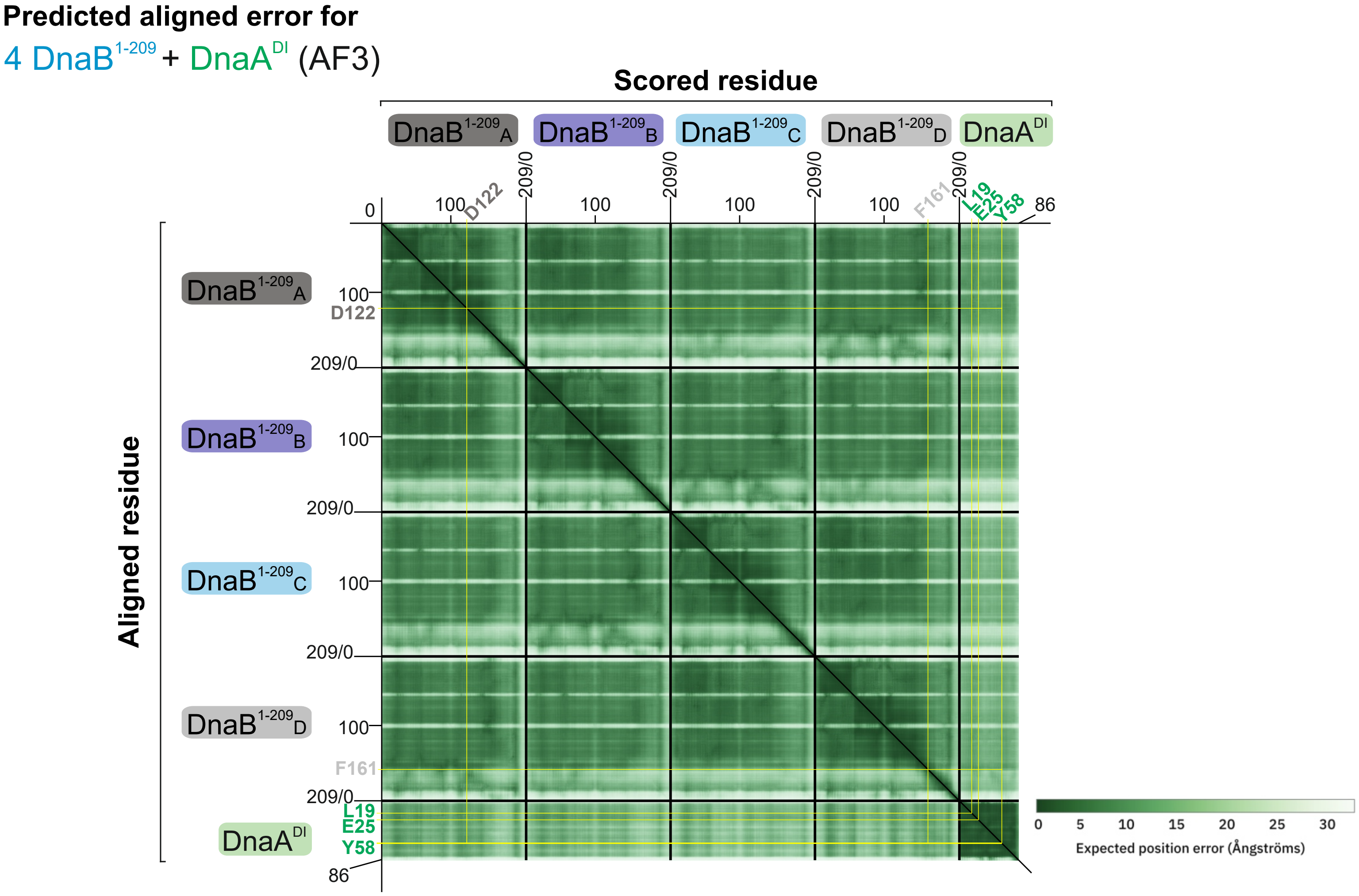

Figure S10. Predicted alignment error for AlphaFold3 model of DnaB-DnaA interface

AlphaFold3 was used to generate a structural model of four DnaB$^{1\text{-}209}$ chains with one DnaA^{DI}. The model served as the basis for fitting DnaA^{DI} into the DnaB cryo-EM interface shown in **Figures 5C-D**. The predicted alignment error (PAE) for the AlphaFold3 model is shown here, with DnaB$^{1\text{-}209}$ chains and DnaA^{DI} indicated. The PAE viewer, positioned chain A and D at proximity of DnaA^{DI}. Selected critical residues are indicated.

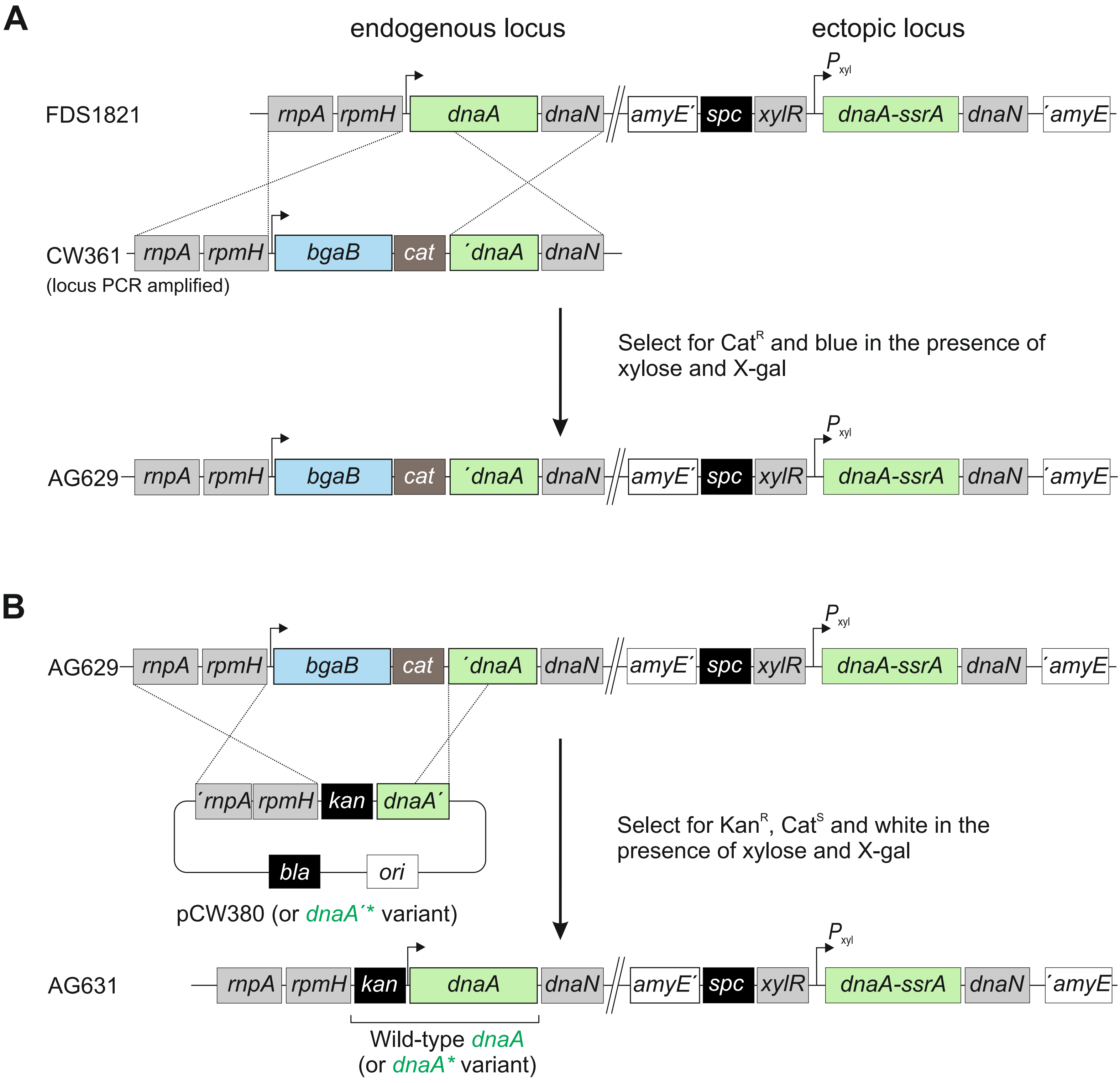

Supplementary Figure S11

Figure S11. Functional analysis of DnaA domain I variants via genetic complementation in *B. subtilis*

(**A**) Construction of the recipient strain AG629 for blue/white screening to introduce amino acid substitutions in DnaA Domain I. A xylose-inducible *dnaA-ssrA* allele (ectopic) was inserted at the *amyE* locus, alongside a spectinomycin resistance cassette, *xylR* and *dnaN*. The endogenous *dnaA* locus was partially deleted (retaining domains III–IV), and the *bgaB* gene (encoding a β-galactosidase, controlled by *Pveg*), integrated along with a chloramphenicol resistance cassette (Cat^R). The final strain, AG629, was selected by plating on Cat^R medium supplemented with xylose and X-gal, and selecting for a blue colony. (**B**) Schematic of DnaA domain I blue/white screening assay. AG629 was transformed with pCW380 (or a derivative carrying a domain I *dnaA'** variant, obtained via PCR site-directed mutagenesis) containing a DNA-segment corresponding to *dnaA* residues 1–249 (Domains I–II and III partial), the upstream flanking region of endogenous *dnaA*, and a kanamycin resistance cassette (Kan^R). Successful homologous recombination at the endogenous *dnaA* locus was selected by plating on Kan^R plate supplemented with xylose and X-gal, followed by screening for white colonies. White colonies were verified to be chloramphenicol sensitive (Cat^S) and spectinomycin resistant (Spc^R) on xylose-supplemented medium.

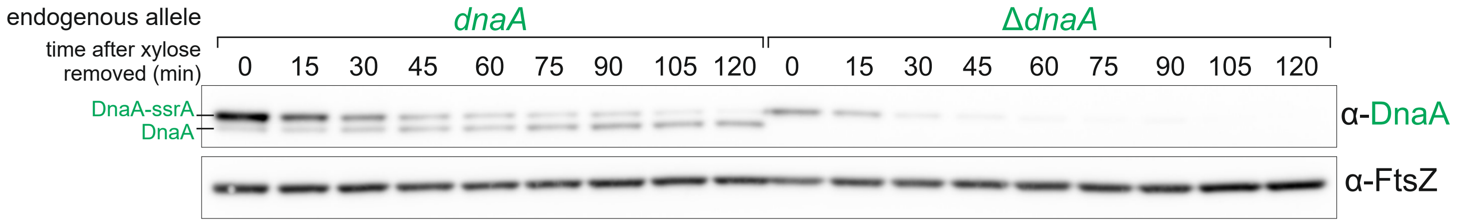

Figure S12. Immunoblot analysis of the inducible *dnaA-ssrA* in controls strains

Immunoblot of DnaA variants following depletion of DnaA-ssrA by washing out xylose (samples collected at indicated time points). The tubulin homolog FtsZ was detected as a loading control. Significant degradation of DnaA-ssrA was observed within 60 min.

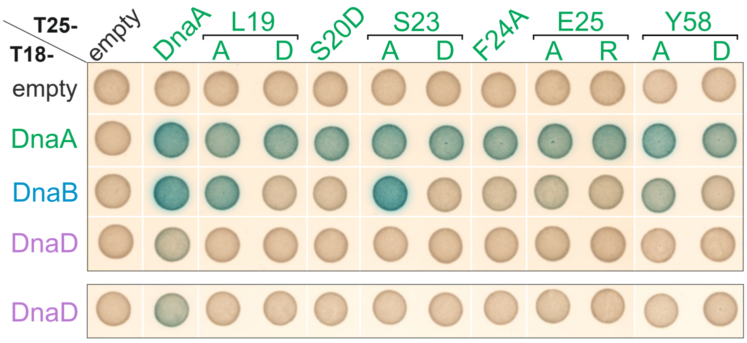

Figure S13. Reduced interaction of DnaD with Domain I DnaA variants

Bacterial two-hybrid assay showing DnaA variants L19A/D, S20D, S23A/D, F24A, E25A/R and Y58A/D decrease the interaction with DnaD. Each black frame corresponds to a distinct plate, with X-gal concentrations of 0.008 % w/v (top) and 0.016 % w/v (bottom). Plates were scanned at 48 h.

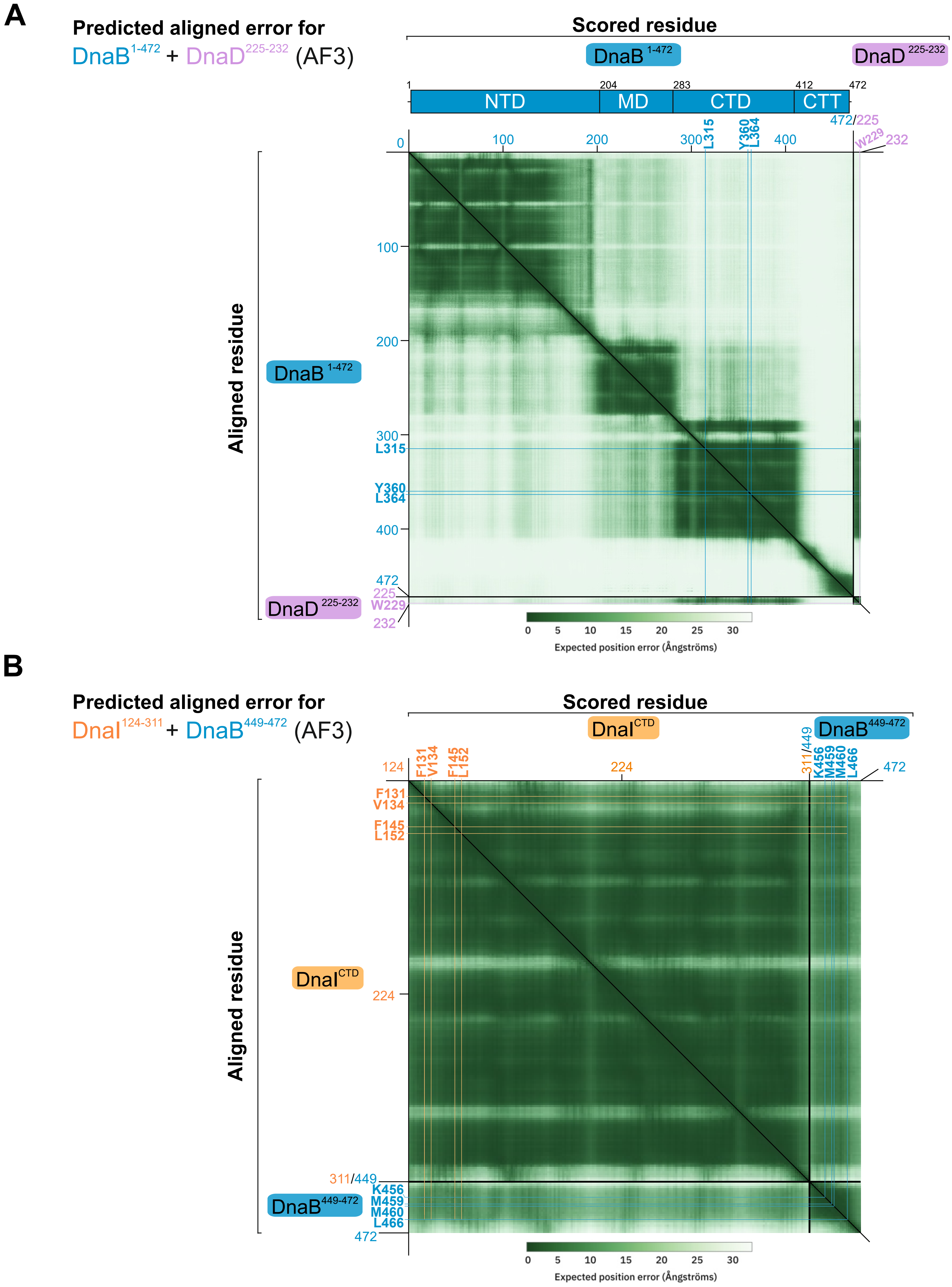

Supplementary Figure S14

Figure S14. Predicted alignment error for AF3 models for PPIs

(**A**) AlphaFold3 was used to generate a structural model of one full length chain of DnaB with the distal end of $DnaD^{CTT}$ (residues 225-232) shown in **Figures 6C-D**. The predicted alignment error for this model is shown here. Selected critical residues are indicated. (**B**) AlphaFold3 was used to generate a structural model of $DnaI^{CTD}$ with the distal end of $DnaB^{CTT}$ (residues 449-472) shown in **Figures 7C-D**. The predicted alignment error for this model is shown here. Selected critical residues are indicated.

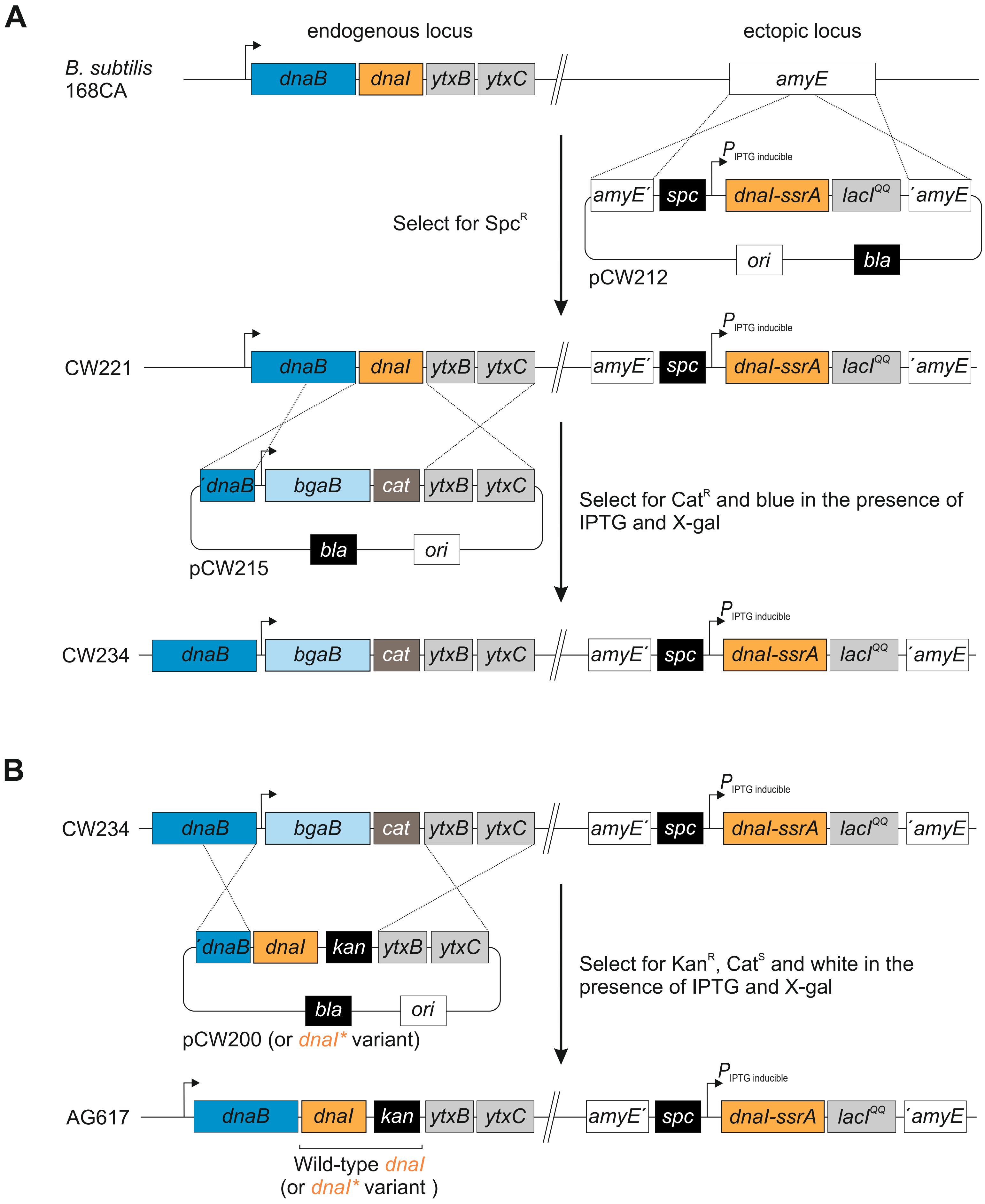

Supplementary Figure S15

Figure S15. Genetic complementation assay for functional analysis of *dnaI* mutants in *B. subtilis*. (**A**) Construction of the recipient strain CW234 for blue/white screening of DnaI variants in *B. subtilis*. An IPTG inducible *dnaI-ssrA* allele (ectopic), a spectinomycin resistance cassette (Spc^{R}) and the regulator *lacI*^{QQ} (encoding LacI$^{Q18M/A109T/W220F}$) were inserted into the *amyE* locus via homologous recombination of plasmid pCW212 and selection for spectinomycin resistance (Spc^{R}). The resulting strain CW221 was then employed to knock out the endogenous *dnaI* copy and substituted with *bgaB* (encodes a β-galactosidase under the control of P_{veg}) and the chloramphenicol cassette via a double recombination event facilitated by plasmid pCW215. The final strain CW234 was isolated by selecting for a blue colony on medium containing IPTG, X-gal, and chloramphenicol (Cat^{R}). (**B**) Schematics of the *dnaI* blue/white screening assay. CW234 was transformed with pCW200 (or a derivative carrying a *dnaI** variant obtained by site-directed mutagenesis) that carries *dnaI* gene, a kanamycin resistance cassette and the homology arms for the endogenous *dnaI* locus. White colonies, resulting from double recombination were selected on medium supplemented with IPTG, X-gal and kanamycin (Kan^{R}). These colonies were verified for chloramphenicol sensitivity (Cat^{S}) and spectinomycin resistance (Spc^{R}) on IPTG supplemented medium.

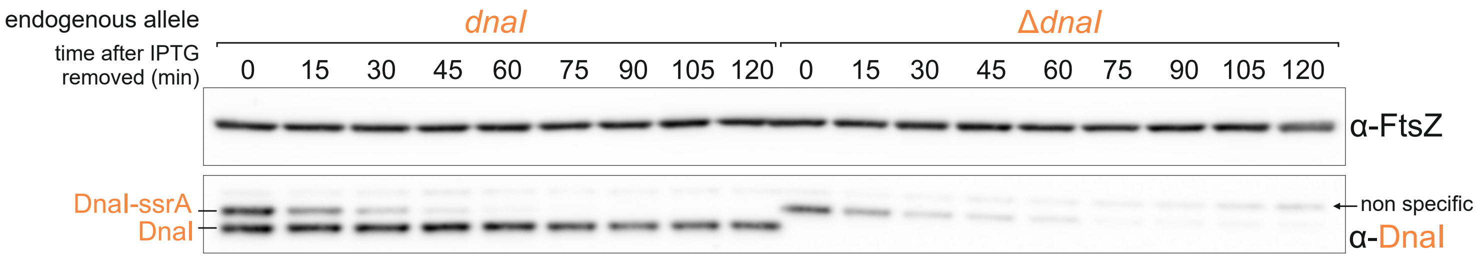

Figure S16. Immunoblot analysis of the inducible *dnaI-ssrA* in controls strains.

Immunoblot of DnaI following depletion of DnaI-ssrA by washing out IPTG. Samples were collected at the indicated time points, and substantial degradation of DnaI-ssrA was observed within 60 min. Equal amounts of each sample were loaded on a separate gel to detect the tubulin homolog FtsZ as a loading control.

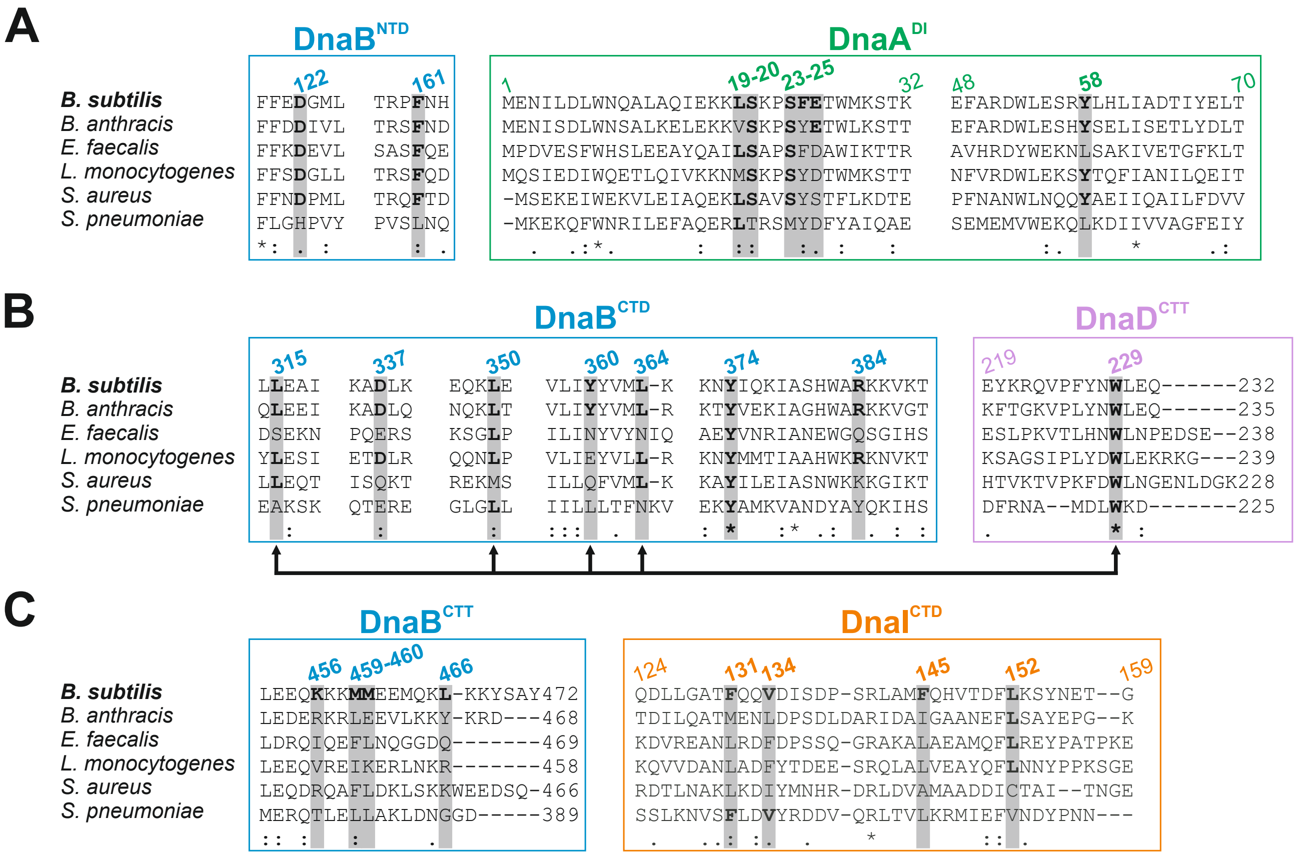

Figure S17. Conservation of DnaB interaction sites in Firmicutes pathogens

Focused amino acid sequence alignments comparing *B. subtilis* DNA replication initiation proteins with homologs from *Bacillus anthracis*, *Enterococcus faecalis*, *Listeria monocytogenes*, *Staphylococcus aureus*, and *Streptococcus pneumoniae*. (A) DnaB^{NTD} and DnaA^{DI}. (B) DnaB^{CTD} and DnaD^{CTT}. (C) DnaB^{CTT} and DnaI^{CTD}.

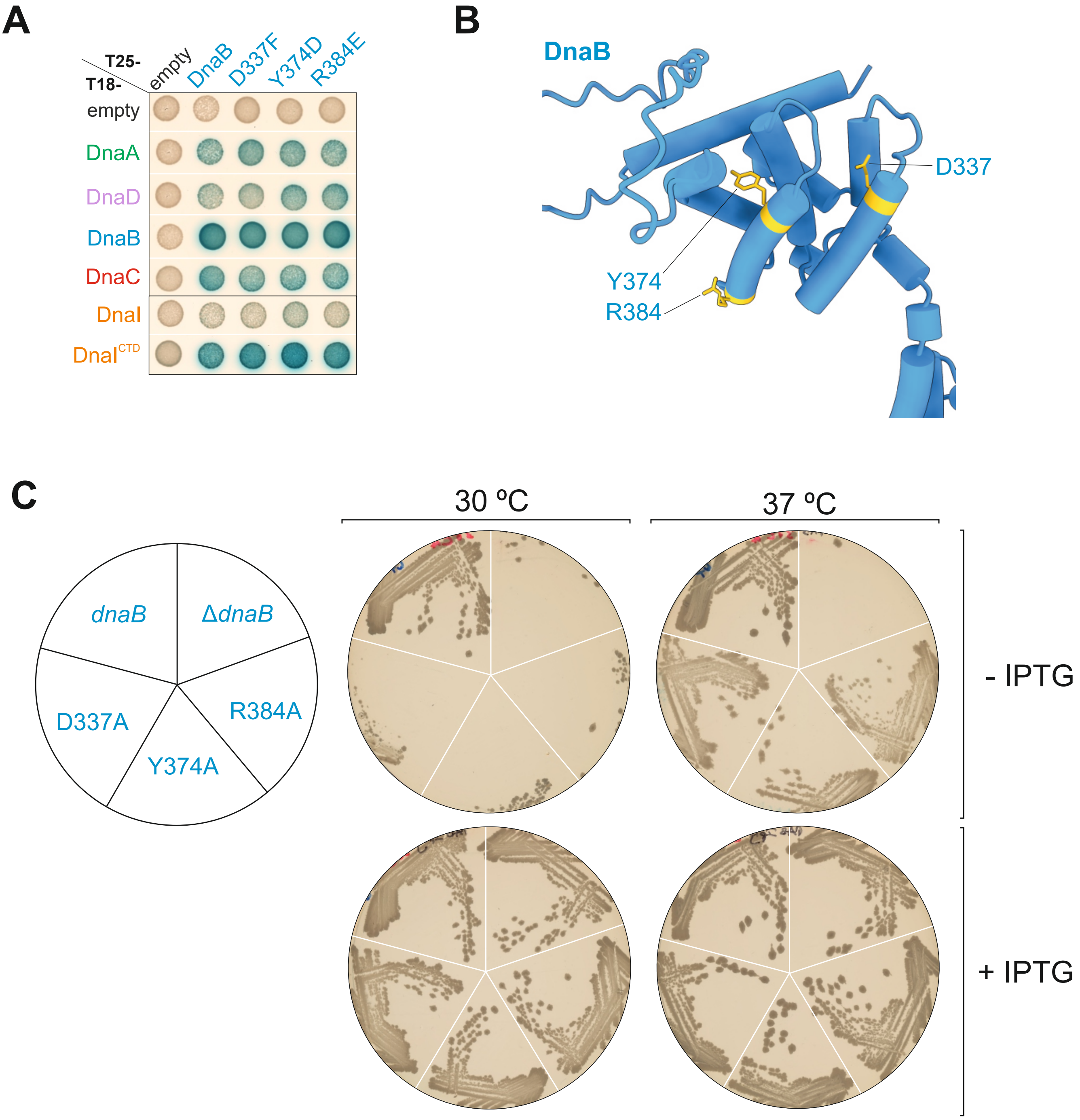

Figure S18. PPI analysis and temperature sensitivity of DnaB D337A, Y374A, and R384A variants
(**A**) B2H assays showing that the DnaB D337F, Y374D and R384E variants interact with DnaA, DnaD, DnaB, DnaC and DnaI^{CTD}. Black frames separate plates containing X-gal at 0.008 % w/v (top) and 0.016 % w/v (bottom) which were scanned at 48 h and 72 h, respectively. (**B**) Representation of one chain of the tetrameric DnaB hybrid model centred on the critical residues D337, Y374 and R384. (**C**) Thermosensitive phenotype identified for DnaB variants D337A, Y374A and R384A. Strains were streaked alongside control strains (wild-type and Δ*dnaB*) on PAB agar supplemented with or without IPTG (0.1 mM). Plates were incubated at 30 or 37 ºC and scanned at 24 h.

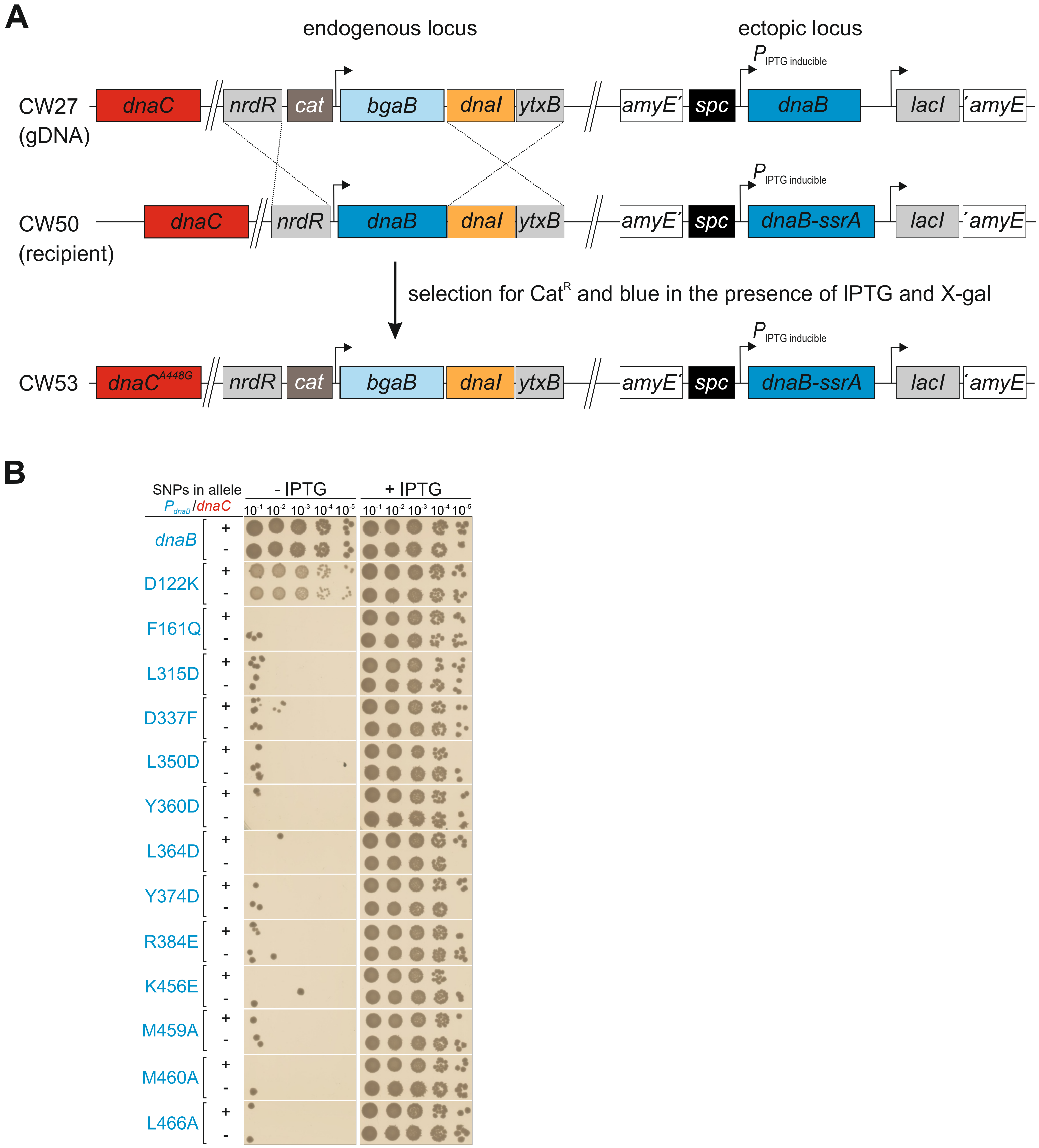

Figure S19. Phenotype of essential DnaB variants without DnaC missense mutation

(**A**) Schematic illustrating the identification of a missense mutation in strain CW53 and its derivatives creating (DnaC^{A448G}). See also **Supplementary Methods**. (**B**) Essential DnaB variants were reconstructed to correct for the *dnaC*^{A448G} mutation and a single base change in the *dnaB* promoter region present in pCW53. Spot titre assays show that DnaB variants with (+) or without (-) these mutations in the P_{dnaB} and *dnaC* alleles grew similarly. All strains were spotted on PAB agar with or without IPTG (0.1 mM), incubated at 37 °C, and scanned at 24 h.
