# Supplementary material for: Bacillus subtilis DnaB forms multiple protein–protein interactions essential for DNA replication initiation
Source: Nucleic Acids Res. 2026 Jul 2;54(12):gkag630. doi: 10.1093/nar/gkag630 (PMC13326638; doi:10.1093/nar/gkag630)
Supplement: gkag630_Supplemental_Files [file gkag630_supplemental_files.zip › Guyet_SupplementalTablesMethods_20260529_clean.docx]

**TITLE:**

***Bacillus subtilis* DnaB forms multiple protein-protein interactions essential for DNA replication initiation**

**Supplementary Tables**

**Table S1.** Oligonucleotides used in this study

**Table S2.** Quick change alanine oligonucleotides used in this study

**Table S3.** Plasmids used in this study

**Table S4.** Bacterial Two-Hybrid plasmids used in this study

**Table S5.** Strains used in this study (excluded DnaB alanine variants)

**Table S6.** *B. subtilis* DnaB alanine variant strains

**Table S7.** Automated growth of DnaB alanine variants grown in PAB medium for 22 h (used to extract 2-8 h growth data for Figure 2D)

**Table S8.** Origin per terminus ratio of each strain analysed by Marker Frequency Analysis, detailed for each biological replicate.

**Table S9.** Numbers of origins, nucleoids and cells per biological experiment for each strain analysed.

**Table S10.** DnaB cryo-EM image processing

**Table S11.** Sequences of protein homologs

**Table S12.** Sequence alignments of DnaB homologs

**Table S13.** Sequence alignments of DnaA homologs

**Table S14.** Sequence alignments of DnaD homologs

**Table S15.** Sequence alignments of DnaI homologs

**SUpplementaRY methods**

**Plasmids construction**

Plasmids were primarily constructed via PCR-based *in-vitro* mutagenesis, either using the NEBuilder HiFi DNA assembly kit (NEB) or by site-directed mutagenesis (quick change). Details of primer pairs, DNA template, parent plasmid and cloning approach used to obtain specific plasmids are indicated in **Supplementary Tables S3** and **S4**. PCR products were amplified with Q5 High-Fidelity DNA polymerase (NEB, also used for sequencing) or PrimeSTAR GXL DNA polymerase (Takara, difficult amplifications) using primers pairs that carried an overlap region (at least 10 bp) to introduce substitutions, deletions or insertions at loci of interest. For insertions, purified PCR-amplified inserts were used as ‘megaprimer’ in a quick change reaction using parent plasmids as template (1). PCR products for *in-vitro* mutagenesis were *Dpn*I (NEB) digested for 3 hours, inactivated, purified and transformed into either *E. coli* CW198 (for *Bacillus* *subtilis* integration plasmids) or DH5⍺. Colony PCRs were performed with GoTaq G2 DNA polymerase (Promega) using primers annealing outside homology regions. Construction of plasmids that required either multi-step assembly or a different cloning method are detailed below.

Bacterial two-hybrid (B2H) plasmids pHM648, pHM650, pHM654 and pHM656 were obtained by restriction cloning of *Asp*718I*-Bam*HI digested inserts (amplified from *B. subtilis* 168CA genomic DNA, gDNA) and digested parent plasmids followed by ligation via T4 DNA ligase (NEB).

pCW31 harbours *nrdR dnaB-dnaI-ytxB* and was obtained by PCR assembly of a pBR322 plasmid backbone amplified with oCW121-oCW122 and a 3.6 kb PCR fragment amplified with oCW119-oCW120 from *B. subtilis* 168CA gDNA.

pCW53 harbours *nrdR* *kan dnaB-dnaI-ytxB*, was used for *B. subtilis* integration at the endogenous *dnaB* locus and employed as template for site-directed mutagenesis. pCW53 was obtained by PCR assembly of a kanamycin resistance cassette (PCR-amplified with oCW166-oCW167 using HM1108 gDNA as template) with PCR-amplified pCW31 using primers oCW168-oCW169. Note that pCW53 harbours a single nucleotide polymorphism (SNP) upstream the *dnaB* start codon annotated *P^SNP^* in genotypes (G to T mutation within the *dnaB* promoter at location ^-67^CATTT**T^SNP^**ATAGA^-57^ with respect to DnaB start codon).

pCW63 harbours *nrdR-cat-P_VEG_-bgaB-dnaI-ytxB* and was obtained after sequential plasmid construction. First, pCW44 was obtained by PCR assembly of the following PCR products: *nrdR* locus (left-homology) and *dnaB-dnaI-ytxB* locus (right-homology) amplified from *B. subtilis* 168CA gDNA with primers pairs oCW119-oCW141 and oCW120-oCW140, respectively; chloramphenicol cassette amplified with oCW138-oCW139 from pHM327 (2), and pBR322 backbone amplified with oCW121-oCW122. Second, pCW62 was obtained by PCR assembly of *bgaB* amplified with oCW143-oCW211 from CW197 gDNA (3) and part of the pCW44 backbone amplified with oCW144-oCW212. Finally, pCW63 was then obtained by quick-change mutagenesis using overlapping primers oCW213-oCW214 to amplify pCW62 and integrate *P_VEG_* upstream of *bgaB*.

pCW110, an integration plasmid carrying the IPTG-inducible *dnaB-ssrA* cassette used for ectopic complementation at *amyE*, was obtained via multi-step assembly. First, pCW05 was generated via NEBuilder Hifi assembly of *dnaB* including endogenous ribosome binding site amplified from *B. subtilis* 168CA gDNA using primers oCW040-oCW41 and the linearised pDR111 plasmid backbone (*Hind*III-*Sph*I restriction enzyme digestion) . Second, pCW64 was generated via quick change mutagenesis to integrate the *P_VEG_* promoter upstream of *lacI* using oCW215-oCW216 and pCW05 as template. Third, pCW110 was generated by quick change mutagenesis to fuse an *ssrA* degradation tag to the 3*'*-end of *dnaB* using oCW330-oCW331 and pCW64 as template. This ssrA degron corresponds to the peptide sequence AANDENYSENYALGG used previously (3,4), as well as for DnaA and DnaI C-terminus protein fusions employed in this study.

pCW193, an integration plasmid carrying the IPTG-inducible *dnaI-ssrA* cassette, was obtained by PCR assembly of the endogenous *dnaI* locus amplified with oCW535-oCW536 using *B. subtilis* 168CA gDNA as template and a section of pCW163 (3) amplified with oCW537-oCW538 (i.e. *dnaD* omitted).

pCW212, the integration plasmid carrying the IPTG-inducible *dnaI-ssrA* cassette used for ectopic complementation at *amyE,* was obtained by quick change mutagenesis to insert three nucleotides between the ribosome binding site and start codon of *dnaI* using oCW578-oCW579 and pCW193 as template (this reduces leaky protein expression in uninduced conditions).

pCW215, an integration plasmid carrying *′dnaB P_VEG_-bgaB cat ytxB ytxC,* was obtained by PCR assembly of the *P_VEG_-bgaB cat* locus amplified with oCW495-oCW496 from CW197 gDNA (3) and a section of pCW200 amplified with oCW493-oCW494 (*i.e.* *dnaI* and kanamycin cassette omitted).

pCW200 harbours *'dnaB-dnaI kan ytxB ytxC*, was used for *B. subtilis* integration at the endogenous *dnaI* locus and employed as template for site-directed mutagenesis. pCW200 was obtained by PCR assembly of the following overlapping fragments: the *'dnaB-dnaI* (left-homology) and *ytxB-ytxC* (right-homology) loci amplified from *B. subtilis* 168CA gDNA using oCW270-oCW408 and oCW553-oCW554, respectively; a kanamycin cassette amplified from pCW53 using oCW267-oCW556, and a pBR322 plasmid backbone amplified with oCW409-oCW555.

pCW380 harbours *rnpA' rpmH kan incA incB dnaA'*, was used for *B. subtilis* integration at the endogenous *dnaA* locus and employed as template for site-directed mutagenesis. pCW380 was obtained by PCR assembly of the following overlapping fragments: the *rnpA'-rpmH* (left-homology) and *incA-incB-dnaA'* (right-homology) loci amplified from *B. subtilis* 168CA gDNA with oCW943-oCW947 and oCW975-oCW976, respectively; a kanamycin cassette amplified from pCW53 with oCW942-oCW974, a pBR322 plasmid backbone amplified with oCW949/950 (5), and a 1.5 kb fragment amplified with oCW1020-oCW1021 from pMAD (6).

pSF15 was used for expression of His_14_-SUMO-DnaB and was obtained by PCR assembly of the pSF14 plasmid backbone (3) amplified with oSF33-oSF162 (*dnaA* locus omitted) followed by *Dpn*I digestion , and the *dnaB* locus amplified with oSF29-oSF165bis from *B. subtilis* 168CA gDNA.

***B. subtilis* strain construction**

Strains are listed in **Supplementary Tables S5** and **S6.**

CW53, the IPTG-dependent *ΔdnaB* recipient blue strain used to reintroduce DnaB variants in *B. subtilis*, was generated via sequential transformations. First, CW17 was obtained by introducing pCW64 into *B. subtilis* 168CA and selecting for spectinomycin resistance (Spc^R^), yielding an IPTG-inducible *dnaB* complementation system. Second, CW27 was generated by introducing pCW63 into CW17 and selecting for chloramphenicol resistance (Cat^R^) in the presence of X-gal (0.008 % w/v) and IPTG (1 mM), thereby replacing the endogenous *B. subtilis* *dnaB* copy by a *cat::(P_VEG_-bgaB)* cassette yielding blue colonies. Third, CW50 was obtained by introducing pCW110 into *B. subtilis* 168CA and selecting for Spc^R^, resulting in an IPTG-inducible *dnaB-ssrA* complementation system. Finally, CW53 was obtained by transforming CW27 gDNA into CW50 and selecting for Cat^R^ associated with blue pigmentation in presence of IPTG (1 mM) and X-gal (0.008 % w/v). During this project, CW53 was found to carry the missense mutation *dnaC^A448G^* discussed at the end of this section.

Reintroduction of *B. subtilis* DnaB variants at the endogenous locus of the IPTG-dependent *ΔdnaB* strain (CW53) was obtained by growing CW53 competent cells for one additional hour prior to starvation in the presence of IPTG (1 mM). Transformations were performed using 800 ng of recombinant DNA (**Supplementary Table S3**) and incubated at 37 °C with vigorous shaking for at least 2 h. After brief centrifugation, the entire transformation reaction was spread onto a single kanamycin plate containing IPTG (1 mM) and X-gal (0.008% w/v), incubated at 37 °C for 24 h, then placed for 24-40 h at room temperature before blue/white screening. For each mutant, three to four white colonies were streaked onto kanamycin plates containing IPTG (1 mM) and PAB agar for rapid phenotype analysis. Selected colonies were validated by confirming chloramphenicol sensitivity (Cat^S^) and Spc^R^ in presence of IPTG (1 mM). After 24 h at 37 °C, colony phenotypes on PAB agar were classified as: (i) no change compared to wild-type, (ii) reduced colony size, (iii) transparent, (iv) sick/heterogeneous, or (v) lethal phenotype (often with suppressors). Genomic DNA from variants with abnormal phenotype was extracted, and endogenous and ectopic *dnaB* copies were PCR-amplified prior to validation via sequencing. DnaB alanine substitutions and additional variants are listed in **Supplementary Tables S6** and **S5**, respectively.

To integrate a dual fluorescence system into DnaB variant genetic backgrounds, strain CW425 (*yycR::erm(tetO^array48^)*) was generated by transforming *B. subtilis* 168CA with BWX721 gDNA (7) by selection for erythromycin resistance (Erm^R^), and strains were verified by validating sensitivity to spectinomycin (Spc^S^) and kanamycin (Kan^S^). Similarly, BWX2006 gDNA (7) was used to create strains HM1846 (*sacA::cat(hbs-mgfp*)) and CW371 (*ycgO::phleo*(*P_ftsW_-tetR-mCherry*) after selection on appropriate antibiotics: chloramphenicol resistance (Cat^R^) with erythromycin and phleomycin sensitivity (Erm^S^ Phleo^S^); and phleomycin resistance (Phleo^R^) with Erm^S^ and Cat^S^, respectively. These three loci were sequentially integrated into CW381 to produce strain CW528 (*amyE::tet(P_HSA+1T_-dnaD^ECssrA-LGG^-lacI^Q18M/A109T/W220F^) yycR::erm(tetO^array48^) ycgO::phleo(P_ftsW_-tetR-mCherry) sacA::cat(hbs-mgfp)*) (3). To create the equivalent *dnaB* background strain (AG523), gDNA from CW50 (*amyE::spec(P_HYSPANK_-dnaB^ECssrA-LGG^-P_VEG_-lacI)*) was transformed into CW528 by selecting for Spc^R^ and confirming tetracycline sensitivity. Finally, AG523 was transformed with pCW53 (wild-type *dnaB*) or gDNA from existing DnaB variants (**Supplementary Tables S5-S6)**. Mutants were selected on kanamycin plates supplemented with IPTG (0.1 mM), restreaked on the same medium, and validated by verifying Spc^R^, Cat^R^, Erm^R^ and Phleo^R^ in the presence of IPTG (0.1 mM). Phenotypes of resulting wild-type DnaB (AG551) or DnaB variants (AG527 to AG529, AG541 to AG544, AG547 to AG550, AG660 and AG662) were confirmed on PAB agar in the absence of IPTG and sequence identity of the endogenous *dnaB* locus validated by sequencing.

AG629, the xylose-dependent *ΔdnaA* recipient blue strain used to reintroduce DnaA variants in *B. subtilis*, was obtained via sequential transformations. First, FDS1352 (*amyE::spc(xylR P_xyl_-dnaA-dnaN)*) was generated by transforming TR672 gDNA into 168CA (8) with selection for Spc^R^ and validation of Kan^S^. Correct integration at *amyE* was validated by colony PCR using oFDS853-oFDS854. Second, FDS1821 (*amyE::spc(xylR P_xyl_-dnaA^EcssrA-LGG^-dnaN)*) was generated by fusing an *ssrA* degron to the 3’-end of *dnaA* via NEBuilder HiFi assembly of two PCR fragments amplified from FDS1352 gDNA using oFDS490-oFDS2512 and oDS489-oFDS2510, respectively, and assembly products were PCR-amplified using oFDS900-oFDS902 prior to transformation in *B. subtilis* 168CA followed by a gDNA backcross by selecting for Spc^R^. Sequence identity at the *amyE* locus was validated by sequencing PCR products amplified from gDNA using oFDS852-oFDS853. Third, an NEBuilder HiFi DNA assembly product (pCW261) was obtained by combining three PCR products: *dnaA* locus left and right-homologies amplified from *B. subtilis* 168CA gDNA with oCW652-oCW722 and oCW724-oCW725, respectively, and a *P_VEG_*-*bgaB-cat* cassette amplified using oCW649-oCW723 from CW197 gDNA (3). *In-vitro* assembly of pCW261 was then PCR-amplified with oCW722-oCW725 and transformed into CW59 by selecting for Cat^R^ in presence of X-gal (0.008 % w/v) and xylose (1 % w/v), yielding the blue strain CW361. Finally, AG629 (*amyE::spc(xylR P_xyl_-dnaA^ECssrA-LGG^-dnaN) ΔdnaA::cat(P_VEG_-bgaB dnaA^DIII/IV^*) was generated by transforming recombinant DNA amplified from CW361 with oFDS405-oFDS412 into FDS1821 and selecting for Cat^R^ associated with blue pigmentation in the presence of X-gal (0.008 % w/v) and xylose (0.5 % w/v) . Strain identity was validated by sequencing PCR products amplifying endogenous and ectopic copies of *dnaA* from gDNA using oFDS405-oFDS412 and oFDS852-oFDS853*,* respectively.

Reintroduction of wild-type and DnaA variants at the endogenous *dnaA* locus of the xylose-dependent *ΔdnaA* strain (AG629) was performed using the same procedure as detailed for generation of DnaB variants (see preparation of competent cells, recovery, antibiotic selection and phenotype verification) using xylose (0.5 % w/v) instead of IPTG and 800 ng of recombinant plasmid pCW380 or derivatives (**Supplementary Table S3**). Resulting wild-type (AG631) and DnaA variant strains (AG632 to AG642) identity was validated by sequencing PCR products amplifying endogenous and ectopic copies of *dnaA* from gDNA using oFDS405-oFDS412 and oFDS852-oFDS853*,* respectively.

CW234, the IPTG-dependent *ΔdnaI* recipient blue strain used to reintroduce DnaI variants in *B. subtilis*, was constructed in two steps**.** First, CW221 was generated by transforming pCW212 into *B. subtilis* 168CA by selecting for Spc^R^ and the absence of α-amylase activity was validated on solid media containing starch, yielding an ectopic IPTG-inducible *dnaI-ssrA* complementation system. Second, CW234 was generated by transforming pCW215 into CW221 with selection for Cat^R^ in the presence of IPTG (0.1 mM) and X-gal (0.008 % w/v), resulting in replacement of the endogenous copy of *dnaI* by a *bgaB* cassette yielding blue colonies.

Reintroduction of wild-type and DnaI variants at the endogenous *dnaI* locus of the IPTG-dependent *ΔdnaI* strain (CW234) was performed using the same procedure as detailed for generation of DnaB variants (see preparation of competent cells, recovery, antibiotic selection and phenotype verification) using 400 ng of recombinant plasmid pCW200 or derivatives. Resulting wild-type (AG617) and DnaI variant strains (AG572, AG575, AG599, AG665, AG668, AG687, AG688 and AG694) identity was validated by sequencing PCR products amplifying endogenous and ectopic copies of *dnaI* from gDNA using oFDS861-oFDS1976 and oFDS852-oFDS853, respectively. The DnaI^F145D^ variant (AG694) was obtained after incubating transformation plates at 30 °C for 24 h. Incubation at 37 °C would otherwise yield heterogenous colony size on kanamycin plates containing IPTG (0.1 mM), for which sequencing identified that both ectopic and endogenous copies of *dnaI* would harbour the *dnaI^F145D^* allele.

Critical DnaB mutants were reconstructed to remove two single-nucleotide polymorphisms (SNPs) present in CW53 (**Supplementary Figure S19A**). For each variant, an NEBuilder HiFi DNA assembly reaction was performed with two PCR products: one obtained by amplification of *gapB-speD-nrdR-kan-P_dnaB_* upstream of the endogenous copy of *dnaB* from AG02 gDNA with oFDS859-oAG677; the other via amplification of *P_dnaB_-dnaB*-dnaI* with oAG676-oFDS1515 using gDNA from DnaB variant strains as template (*dnaB**). Primers oAG676-oAG677 had an overlap that reconstituted the endogenous *P_dnaB_* and NEBuilder HiFi DNA assembly products were subsequently amplified by PCR with oFDS505-oFDS754. Each PCR product was transformed into CW50 competent cells and transformants were selected on kanamycin plates in the presence of IPTG (0.1 mM). Genomic DNA from resulting strains (AG705 to AG711, AG714 to AG720, AG739, and AG743) was extracted and endogenous *dnaB,* *dnaC* and *amyE* loci were PCR-amplified using oFDS861-oFDS1976, oAG484-oAG487, and oFDS852-oFDS853, respectively. PCR sequencing via Plasmidsaurus validated correct identity of reconstructed strains. Spot titre analyses of reconstructed DnaB variants showed that important DnaB residues remain critical in this background (**Supplementary Figure S19B**).

**Supplemental references**

S1. Miyazaki, K. (2011) MEGAWHOP cloning: a method of creating random mutagenesis libraries via megaprimer PCR of whole plasmids. *Methods Enzymol*, **498**, 399-406.

S2. Scholefield, G., Errington, J. and Murray, H. (2012) Soj/ParA stalls DNA replication by inhibiting helix formation of the initiator protein DnaA. *EMBO J*, **31**, 1542-1555.

S3. Winterhalter, C., Stevens, D., Fenyk, S., Pelliciari, S., Marchand, E., Soultanas, P., Ilangovan, A. and Murray, H. (2023) SirA inhibits the essential DnaA:DnaD interaction to block helicase recruitment during *Bacillus subtilis* sporulation. *Nucleic Acids Res*, **51**, 4302-4321.

S4. Griffith, K.L. and Grossman, A.D. (2008) Inducible protein degradation in *Bacillus subtilis* using heterologous peptide tags and adaptor proteins to target substrates to the protease ClpXP. *Mol Microbiol*, **70**, 1012-1025.

S5. Bolivar, F., Rodriguez, R.L., Greene, P.J., Betlach, M.C., Heyneker, H.L., Boyer, H.W., Crosa, J.H. and Falkow, S. (1977) Construction and characterization of new cloning vehicles. II. A multipurpose cloning system. *Gene*, **2**, 95-113.

S6. Arnaud, M., Chastanet, A. and Debarbouille, M. (2004) New vector for efficient allelic replacement in naturally nontransformable, low-GC-content, gram-positive bacteria. *Appl Environ Microbiol*, **70**, 6887-6891.

S7. Wang, X., Montero Llopis, P. and Rudner, D.Z. (2014) *Bacillus subtilis* chromosome organization oscillates between two distinct patterns. *Proc Natl Acad Sci U S A*, **111**, 12877-12882.

S8. Richardson, T.T., Stevens, D., Pelliciari, S., Harran, O., Sperlea, T. and Murray, H. (2019) Identification of a basal system for unwinding a bacterial chromosome origin. *EMBO J*, **38**, e101649.
